# Quaternary structures of *Streptococcus pneumoniae* nucleoside diphosphate kinase: From hexamers to supramolecular assemblies

**DOI:** 10.1002/pro.70735

**Published:** 2026-08-08

**Authors:** Julie Kerboeuf, Paul Nouri, Frédéric Galisson, Laetitia Daury, Marie‐France Giraud, Olivier Lambert, Cédric Orelle, Lionel Ballut, Elise Kaplan, Jean‐Michel Jault, Cécile Gonzalez

**Affiliations:** ^1^ CNRS, UMR 5086, Molecular Microbiology and Structural Biochemistry Universite de Lyon Lyon France; ^2^ CNRS, Bordeaux INP, CBMN, UMR 5248 University of Bordeaux Pessac France

**Keywords:** 3D structure, dodecamer, hexamer, *Kpn*‐loop, mass photometry, nucleoside diphosphate kinase, quaternary structure, *Streptococcus pneumoniae*

## Abstract

The nucleoside diphosphate kinase (NDK) is a key enzyme that controls the balance of nucleotide pools in all living organisms. Beyond this fundamental role, NDKs exert pleiotropic effects in many cellular processes, including cell development, signal transduction, differentiation, tumor metastasis, and gene expression. The quaternary structure of NDK is typically hexameric—organized as a trimer of dimers in eukaryotic cells and in many prokaryotes—though in some species it can also be tetrameric. Here, we report the crystal structure of *Streptococcus pneumoniae* NDK (SpNDK) in its apo state (1.2 Å) and in an adenosine diPhosphate (ADP)‐vanadate‐bound state (3.4 Å). In both structures, SpNDK adopts a hexameric assembly and the fold of each monomer is highly conserved compared to NDKs from other organisms. A notable feature is the extended *Kpn*‐loop, which plays a key role in stabilizing the hexamer. The protein exhibited remarkably high thermal stability (*T*_m_ ~ 76°C). However, mutation of R28 (R28A), which interacts with the *Kpn*‐loop, destabilized the hexamer, lowering the *T*_m_ by >20°C. Unexpectedly, size‐exclusion chromatography and mass photometry revealed that wild‐type SpNDK exists as an equilibrium mixture of hexamers, dodecamers, and higher‐order supramolecular assemblies. Using cryo‐electron microscopy, we solved the three Dimensional (3D) structure of the hexameric state at 2.47 Å resolution, and resolved dodecameric assemblies of the protein. Since the oligomeric state of the NDK influences its cellular function, further investigations will be needed to address the in vivo relevance of these findings, in line with the broad and multifaceted roles of this enzyme family.

## 1 INTRODUCTION

*Streptococcus pneumoniae* is a Gram‐positive bacterium responsible for a broad spectrum of infections, including pneumonia, meningitis, and sepsis (Narciso et al., 2025). While vaccines targeting some *S. pneumoniae* serotypes are available (Weiser et al., 2018), the rising prevalence of antibiotic‐resistant strains poses a significant public health challenge and underscores the urgent need for novel therapeutic strategies (GBD 2021 Antimicrobial Resistance Collaborators, 2024). At the molecular level, targeting specific proteins—particularly enzymes essential for bacterial growth—offers promising avenues to combat *S. pneumoniae* infections. Moreover, proteins that contribute to bacterial virulence represent valuable therapeutic targets for curbing pathogen dissemination during infection. Among these, enzymes involved in nucleotide metabolism, such as nucleoside diphosphate kinase (NDK) (Gupta et al., 2022), are of particular interest. NDKs (*Escherichia coli* 2.7.4.6) are ubiquitous enzymes that catalyze the transfer of the terminal phosphate group from nucleoside triphosphates (NTPs) to nucleoside diphosphates (NDPs). Beyond their pivotal role in maintaining cellular nucleotide homeostasis, NDKs participate in numerous biological processes including DNA replication, signal transduction, and cellular proliferation (Boissan et al., 2018; Schlattner, 2021). In bacteria, NDKs have also been implicated in stress response, biofilm formation, regulation of virulence‐associated pathways, and host cell defense mechanisms (Li et al., 2024; Sun et al., 2013; Yu et al., 2017; Zhang et al., 2020).

NDKs are evolutionarily conserved enzymes present across all domains of life and share a common ferredoxin‐like fold at the monomeric level (Georgescauld et al., 2020). However, their biological assemblies differ depending on the organism. In eukaryotes, including mammals and plants, NDKs typically assemble as hexamers and multiple isoforms exist, up to 10, with locations spanning the cytoplasm, mitochondria, chloroplast, and nucleus (Bolter et al., 2007; Georgescauld et al., 2020). In bacteria, NDKs occur in three main oligomeric forms: hexamers are typical of Gram‐positive bacteria such as *Staphylococcus aureus* (Srivastava et al., 2011) and *Mycobacterium tuberculosis* (Chen et al., 2002); tetramers can be found in some Gram‐negative species like *Escherichia coli* (Moynie et al., 2007); and dimers are observed in others, such as *Halomonas* sp. (Arai et al., 2012). Regardless of their origin, the building block for oligomer formation is always a dimer, with tetramers and hexamers arising from the association of two or three dimers, respectively (Georgescauld et al., 2020).

Here, we present the first x‐ray crystal structure of *S. pneumoniae* NDK (SpNDK) at 1.2 Å resolution. The asymmetric unit of this crystal comprises a single monomer; nevertheless, crystallographic symmetry operations reconstitute the canonical hexamer, consistent with the physiological oligomeric state of the enzyme. Additionally, we determined the structure of SpNDK in complex with ADP and vanadate at 3.4 Å resolution, revealing that the nucleotide binds in a manner analogous to previously solved NDK structures. Importantly, size‐exclusion chromatography and mass photometry uncovered higher‐order assemblies in solution, most prominently 12‐ and 18‐mers, which likely correspond to dimers and trimers of hexamers, respectively. Wild‐type (WT) SpNDK was remarkably thermostable (*T*_m_ ~ 76°C) and this stability results primarily from the *Kpn*‐loop which stabilizes the hexameric assembly of the enzyme. On the other hand, the existence of dodecameric species was further validated by cryo‐electron microscopy (cryo‐EM) analyses. These supramolecular assemblies are affected by the presence of adenosine triPhosphate (ATP)/guanosine triPhosphate (GTP), as emphasized by the use of the catalytic inactive H121A mutant. They have not been previously described for any bacterial enzyme before, and given the diverse cellular functions of NDKs, their presence suggests new, unrecognized regulatory or scaffolding roles.

## 2 RESULTS

### 2.1 Atomic spNDK structures

**FIGURE 1:**
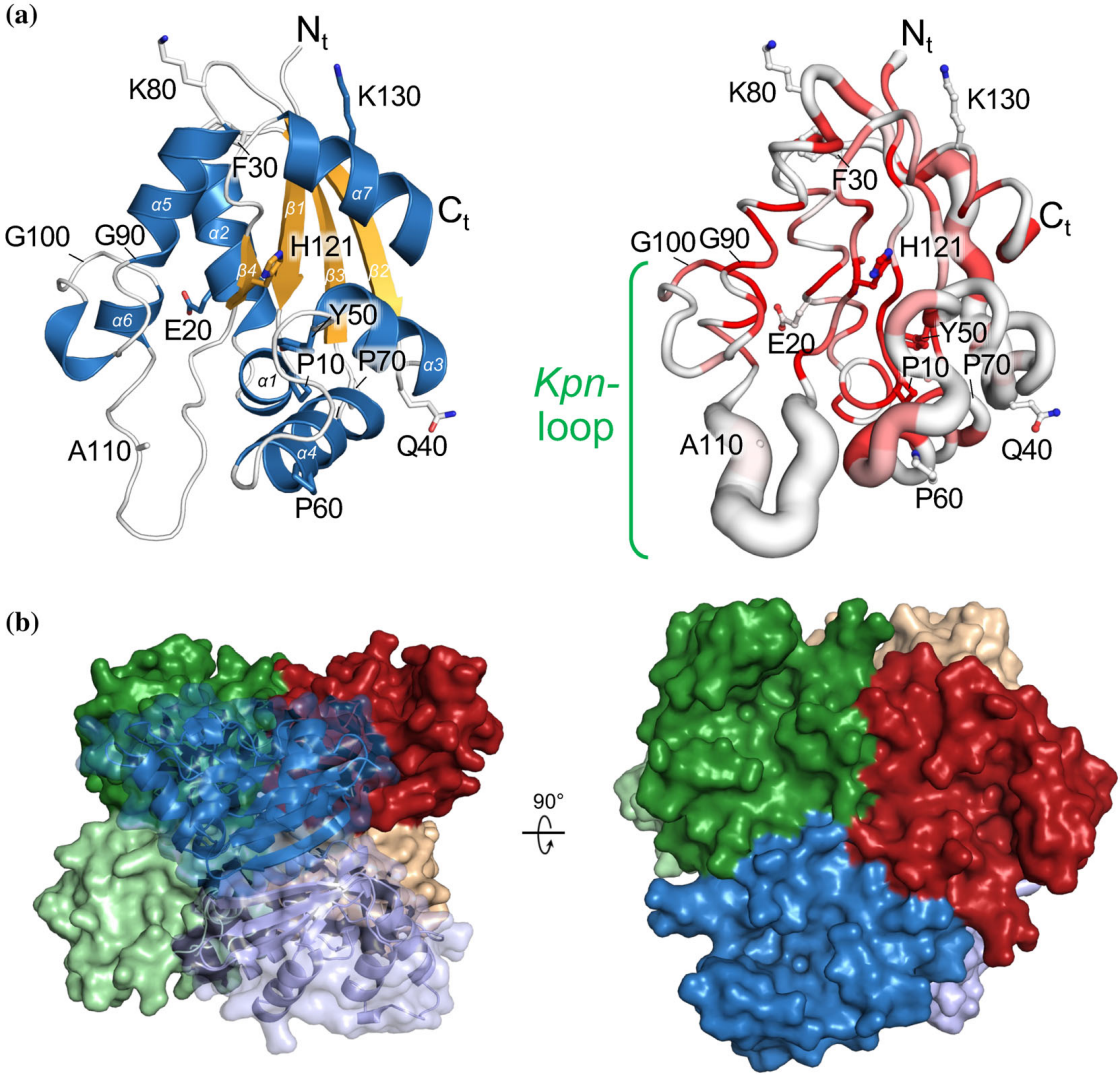
Crystal structure of the *Streptococcus pneumoniae* nucleoside diphosphate kinase (NDK). (a) Cartoon (left) and sausage (right) representation of the apo form of *Streptococcus pneumoniae* NDK (SpNDK). In the sausage representation, the protein is colored according to sequence conservation from white, for non‐conserved residues, to red for strictly conserved residues. Residues numbered at intervals of 10, except the H121 from the active site, are shown as sticks. The tube diameter is proportional to the C_α_ position difference between SpNDK and known structures of homologous NDKs using the PDBAA70 database. The right panel was drawn using ENDscript 2.0 (Robert & Gouet, 2014) and visualized with PyMol. (b) Hexameric assembly of the apo SpNDK reconstructed from crystal symmetry. In the left panel, the dimer (blue shades) is displayed in cartoon representation with a transparent surface overlay.

**FIGURE 2:**
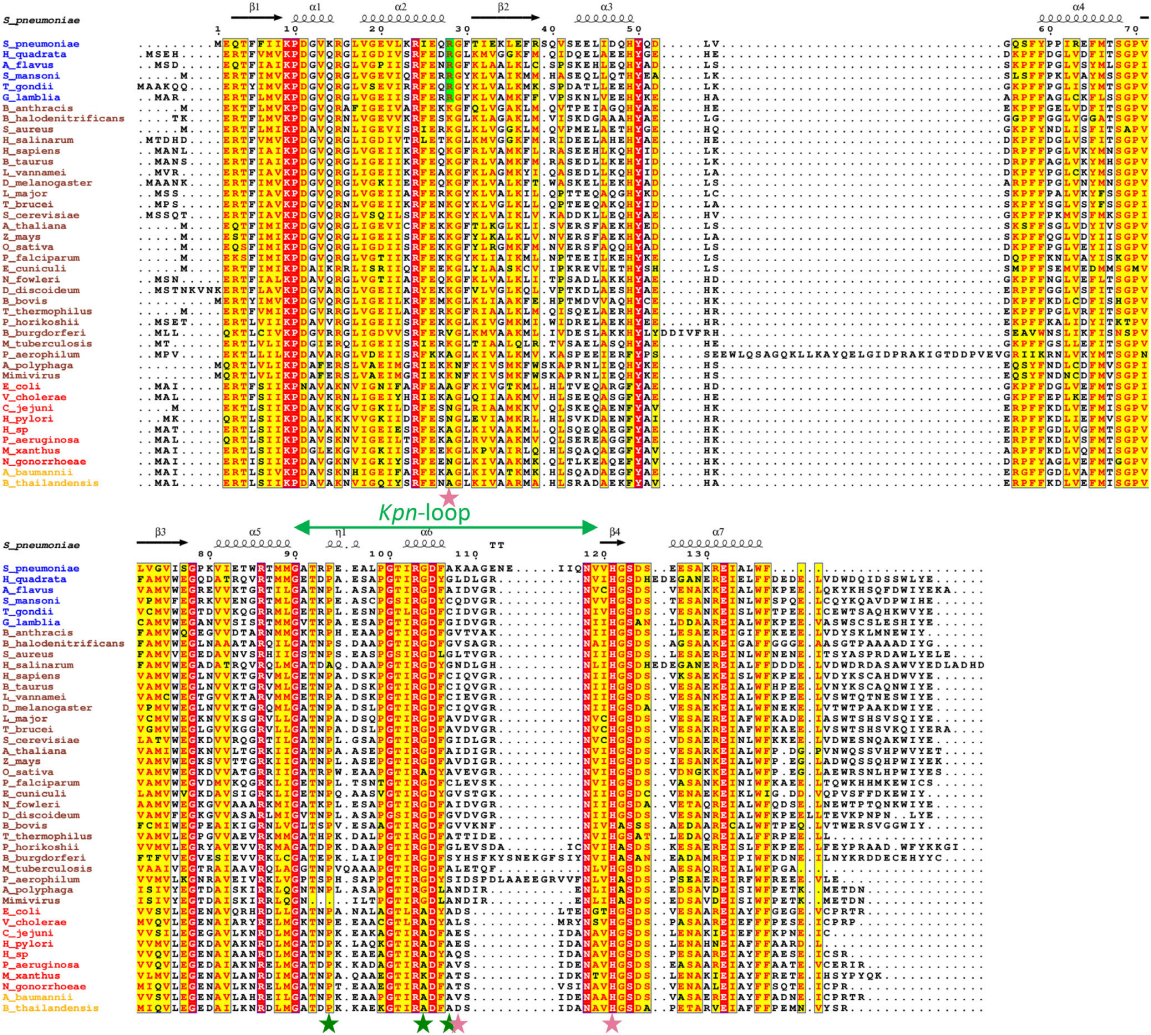
Sequence alignment of nucleoside diphosphate kinase (NDKs) with known 3D structures. NDK sequences are from *Streptococcus pneumoniae* (Protein Data Bank [PDB] 9RVW), *Litopenaeus vannamei* (4UOF), *Thermus thermophilus* (1WKJ), *Schistosoma mansoni* (5IOL), *Drosophila melanogaster* (1NDL), *Aspergillus flavus* (6JOH), *Homo sapiens* (1NUE), *Arabidopsis thaliana* (1U8W), *Toxoplasma gondii* (5BXI), *Bos taurus* (1BHN), *Mycobacterium tuberculosis* (4ANC), *Oryza sativa* (1PKU), *Leishmania major* (3NGR), *Trypanosoma brucei* (4FKX), *Zea mays* (1VYA), *Giardia lamblia* (3R9L), *Saccharomyces cerevisiae* (3B54), *Naegleria fowleri* (5U2I), *Bacillus anthracis* (2VU5), *Staphylococcus aureus* (3Q83), *Acanthamoeba polyphaga (*3EM1), *Vibrio cholerae* (5X00), *Dictyostelium discoideum* (1B99), *Plasmodium falciparum* (1XIQ), *Pyrococcus horikoshii* (2CWK), *Babesia bovis* (3JS9), *Campylobacter jejuni* (3PJ9), *Haloarcula quadrata* (2ZUA), *Halobacterium salinarum* (2AZ3), *Mimivirus* (2B8Q), *Halomonas* sp. (3VGU), *Pseudomonas aeruginosa* (6AES), *Escherichia coli* (2HUR), *Acinetobacter baumannii* (4WBF), *Virgibacillus halodenitrificans* (1NB2), *Borrelia burgdorferi* (4DI6), *Myxococcus xanthus* (1NHK), *Neisseria gonorrhoeae* (5V6D), *Burkholderia thailandensis* (4DUT), *Helicobacter pylori* (6AY1), *Encephalitozoon cuniculi* (3MPD), *Pyrobaculum aerophilum* (1XQI). Species names in blue or brown indicate NDK crystal structures that form hexamers, while those in red correspond to tetrameric assemblies. Names in blue have an Arginine residue in position 28 (boxed in green). Names in orange indicate dimers, with the physiological assembly presumed to be hexameric. The secondary structure of *Streptococcus pneumoniae* NDK (SpNDK) is shown on top of the alignment. The figure was drawn with ESPript 3.0 with strictly conserved residues shown on a red background, and highly similar residues (present in at least 70% of the sequences) displayed on a yellow background (Robert & Gouet, 2014). The residues highlighted by light pink or green stars underneath the sequences have been mutated into Ala in SpNDK (i.e., R28, K108, and H121) or are forming the so‐called “connecting platform” (i.e., P94, G104, and A107) in Figure S4, respectively.

**FIGURE 3:**
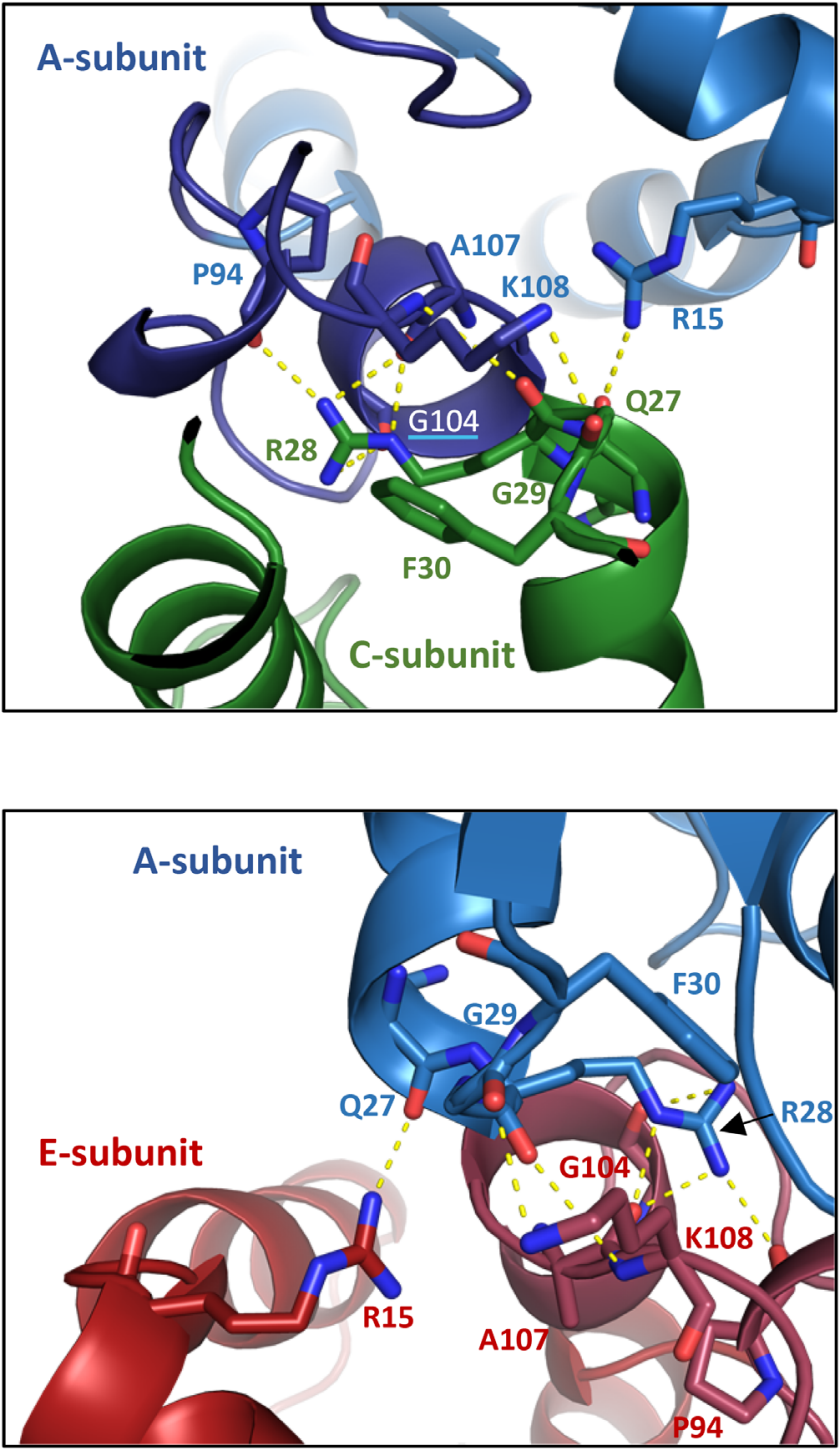
Subunit interface in the hexameric assembly of apo *Streptococcus pneumoniae* nucleoside diphosphate kinase (SpNDK). Residues forming H‐bonds between the A–C subunits (top), and the A–E subunits (bottom) are shown as sticks. The color coding is the same as in Figure 1b, except that the *Kpn*‐loop, including residues P94, G104, A107, and K108 is colored in deep blue in the A‐subunit (top panel) and in raspberry in the E‐subunit (bottom panel).

We used x‐ray crystallography to determine the atomic structures of SpNDK in both its unliganded (apo) form and in complex with ADP and vanadate. Data collection and refinement statistics are presented in Table S2. The apo structure, solved at the very high resolution of 1.20 Å, contains a single monomer in the asymmetric unit (Figure 1a). The overall structure of SpNDK monomer adopts a ferredoxin‐like fold, characterized by an anti‐parallel β‐sheet core (β2‐β3‐β1‐β4) surrounded by eight α‐helices (Figure 1a). As typically observed in NDKs, the catalytic histidine, H121, is located on the β4 strand. Application of crystallographic symmetry reconstructs the NDK homohexamer, arranged as a trimer of dimers (Figure 1b). The contact surface area between two protomers forming a dimer is approximately 600 Å^2^, as calculated with protein interfaces, surfaces and assemblies (Krissinel & Henrick, 2007). This interface primarily involves residues Val18, Gly19, Glu26, Leu35, and Phe37 from both protomers. Within a dimer, the β‐strands of the two monomers align continuously, forming a β‐sheet composed of eight strands in total (Figure 1b). Interestingly, SpNDK has the shortest *C*‐terminal segment among all previously solved NDK structure, ending immediately after the α‐helix 6 (Figure 2). Given the notorious involvement of this region in the hexameric assembly of many NDKs (Georgescauld et al., 2020), including, for instance, the *Borrelia burgdorferi* (Figure S1), *S. aureus* or *Pyrococcus horikoshii* enzymes (Figure S2), this suggests that additional structural elements must contribute to stabilize the hexamer formation in SpNDK. Notably, the *Kpn*‐loop is extended in SpNDK (Figure 2) and is structurally quite divergent as compared to other NDK proteins (Figures 1a and S3). Yet, the structural variation in the *Kpn*‐loop concerns exclusively the second part of the loop, while its first part including the α‐helix 6 is totally conserved (Figure S3). Interestingly, this short α‐helix 6 stacks directly on top of the α‐helix 5 (Figure S3) forming an extended helix that likely constrains the segment between α‐helices 5 and 6 to adopt a similar fold. In SpNDK, this segment of the *Kpn*‐loop strongly contributes to the interaction with the two other dimers forming the hexamer (Figure S1). In particular, Arg28 from the A‐subunit is a major actor providing with its side‐chain four hydrogen bonds with residues Pro94, Gly104, and Ala107 from the *Kpn*‐loop of the neighbor E‐subunit (Figure 3; see the subunit arrangement in Figure S1). These Pro94, Gly104, and Ala107 residues form a “connecting platform” at the subunit interface of SpNDK (Figures 3 and S4) which is well‐conserved both in topology and sequence in many NDKs, including in *E. coli* (i.e., Pro95, Ala105, and Ala108; Figure S4). Yet, despite this conservation, these three residues are only involved in subunit/subunit interaction in hexameric NDKs (Figure S4, panels B–F), whereas in tetrameric NDKs such as *E. coli*, the vicinal subunit is too far away to use this “connecting platform” (Figure S4, panels G and H). Moreover, this platform stabilizes the oligomeric states in various ways depending on the NDK species and the residue facing it on the opposite subunit. Hence, at the position corresponding to Arg28 of spNDK, an Arg or Lys residue is found in archaeal and eukaryal NDKs, whereas in bacterial NDKs, a Lys or smaller side‐chain residue is present at this position (Figure 2). The presence of a Lysine at this position can provide up to three hydrogen bonds through its side chain to mediate a strong interaction with the three residues forming the connecting platform (Figure S4, panels C and D). In addition, a residue from the α‐helix 5 (e.g., His86 in *S. aureus*) provides an additional interaction with the main chain of the Proline residue from the connecting platform. In contrast, when a smaller side‐chain residue is present at the position equivalent to Arg28, for example, Ala in *Pyrobaculum aerophilum* or Val in *B. burgdorferi*, a Lysine residue from the α‐helix 5 of the C‐subunit forms a hydrogen bond through its side chain with the Proline residue of the connecting platform. In conclusion, the connecting platform can play a significant role in stabilizing the hexamer in some NDKs but its implication can be more restricted, reducing to a single hydrogen bond, as observed in the *B. burgdorferi* enzyme (Figure S4, panel F). In SpNDK, Lys108 is also involved in the interaction with the vicinal subunit by providing one hydrogen bond through its side chain with the Gly29 residue of the opposite subunit and in van der Waals distance of Phe30 residue (Figure 3). This Lysine is however located in the beginning of the divergent part of the *Kpn*‐loop and is therefore not conserved in other NDKs (Figure 2). The NDK from *M. tuberculosis* (MtNDK) is another example with a short *C*‐terminus extension (Figure 2), which also form hexamers (Chen et al., 2002). In this case, two residues (ArgR80 and Asp93) are forming a salt bridge to stabilize the physiological assembly (Figure S1). An extended *Kpn*‐loop is also present in two NDKs from *B. burgdorferi* and *P. aerophilum* (Figure S1), both of which assemble as hexamers. In both cases, this loop contributes to the interactions within the hexamer although different residues scattered throughout the loop participate in subunit contacts (Figure S1).

**FIGURE 4:**
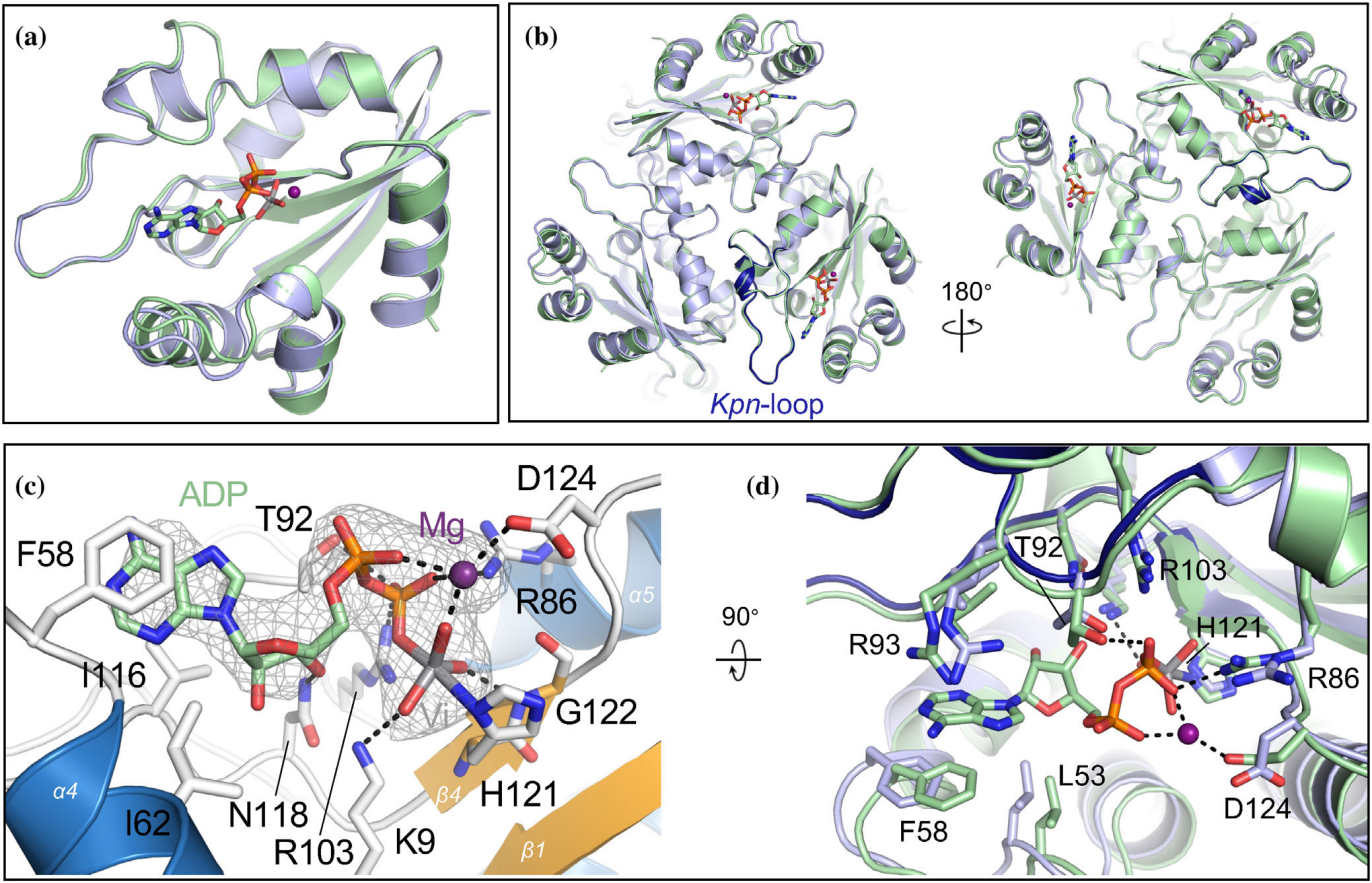
Close‐up view of the ADP‐Vi binding site of *Streptococcus pneumoniae* nucleoside diphosphate kinase (SpNDK). (a) Structural comparison between apo (blue) and ADP‐Vi‐bound form (green) of a SpNDK monomer. (b) Same as A but the overlay is made with the hexameric forms of SpNDK. (c) Close‐up view of the ADP‐Vi binding site of SpNDK. Main chains of interacting residues are in gray sticks. Vanadate (Vi), in trigonal bipyramidal conformation, is covalently bound to the β‐phosphate of ADP (green). A magnesium ion (purple) coordinates the ADP phosphates and the vanadate. The omit map density for the MgADP‐Vi is displayed as a gray mesh, contoured at 3.0 *σ*. (d) The binding of the ADP‐Vi induces a minor reorientation of the side chain of some residues from the nucleotide‐binding pocket.

The crystal structure of the ADP/Vi‐bound form of SpNDK was obtained at a resolution of 3.4 Å, with a hexameric assembly present in the asymmetric unit of the crystal (Movie S1). The binding of nucleotide in the presence of vanadate does not induce a major structural change in the monomer (Figure 4a) or in the hexamer assembly (Figure 4b), and both the apo and holo state models could be aligned with a root mean square deviation (rmsd) of 0.32 Å over 120 C_α_. The residues forming the nucleotide‐binding site are shown in Figure 4c and are either strictly conserved (for Lys9, Arg86, Arg103, Asn118, and His121) or highly conserved (for Phe58, Ile62, Thr92, and Asp124; Figure 2). One notable exception is Ile116, which is located in the extended *Kpn*‐loop of SpNDK. It interacts on one side with the adenine moiety of the bound nucleotide, while Phe58 creates a π‐stacking interaction on the other side with the adenine rings. The binding of nucleotide induced a moderate reorientation of the side chain of some residues in the binding pocket (Figure 4d). Regarding the vanadate, distances and electron density maps suggest that it is bound in a trigonal bipyramidal conformation and is covalently linked to an oxygen atom of the β‐phosphate of ADP (Figure 4c), as also observed in the crystal structure of *B. burgdorferi* NDK (Dumais et al., 2018). However, the limited resolution of our ADP/Vi/NDK complex does not permit ruling out an alternative tetrahedral configuration of vanadate, like the one found in the *S. aureus* enzyme (Srivastava et al., 2011).

**FIGURE 5:**
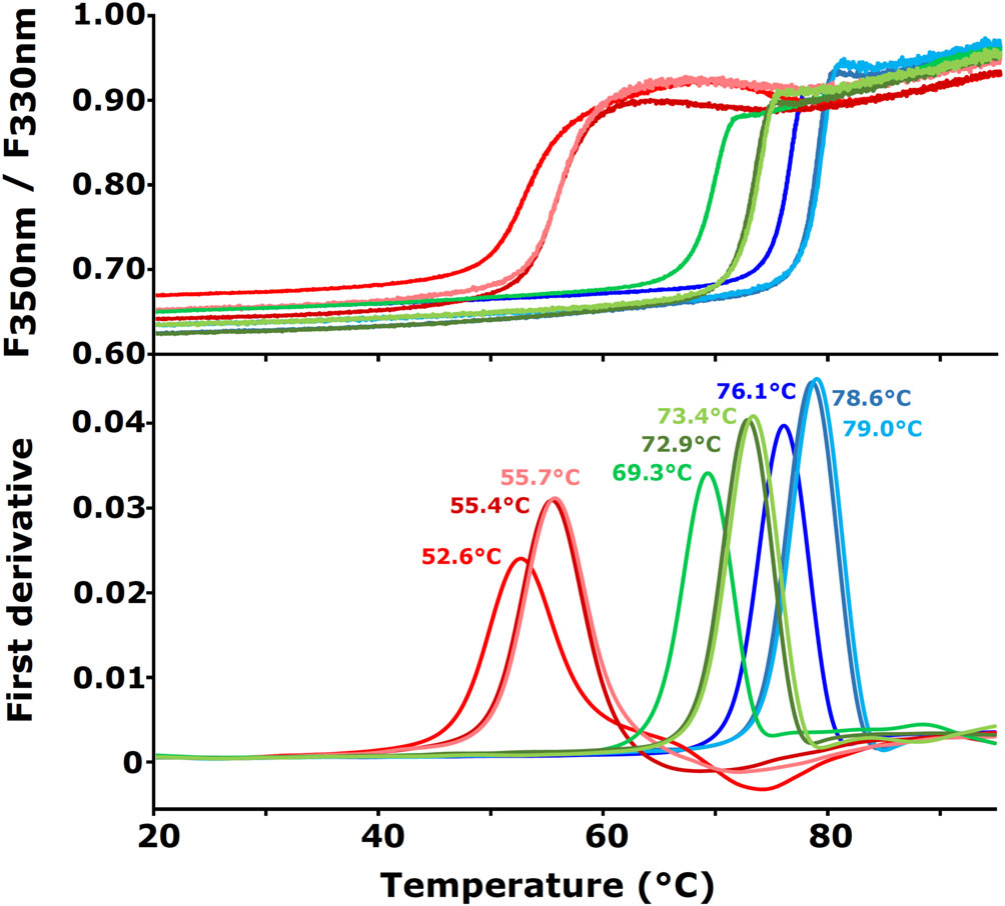
Thermal stability of nucleoside diphosphate kinase (NDK) measured by nanoDSF. Wild‐type *Streptococcus pneumoniae* NDK (SpNDK) (blue‐toned curves), R28A mutant (red‐toned curves) or K108A (green‐toned curves) were preincubated 20 min at 22°C with Mg‐ATP (dark‐blue, dark‐red and dark‐green curves), Mg‐GTP (light‐blue, light‐red and light‐green curves) or without any ligand (blue, red and green curves) prior to gradually increasing the temperature at 1°C.min^−1^. The fluorescence ratio (*F*_350nm_/*F*_330nm_) and its first derivative are shown in the top and bottom panels, respectively. The inflection points of the derivatives, corresponding to the melting temperatures (*T*_m_), are reported in the figure.

The thermal stability of purified SpNDK was assessed by nanoDSF (Figure 5). The enzyme was remarkably stable with a very high melting temperature (*T*_m_ ~ 76°C) which was only moderately further stabilized by nucleotides, Mg‐ATP or Mg‐GTP, with a *T*_m_ increased by ~3°C. This is consistent with the moderate impact of nucleotide on the overall structure of SpNDK (Figure 4d). Because we suspected that this high *T*_m_ was related to the oligomeric state of SpNDK, we mutated the Arg28 or Lys108 into Ala to weaken the quaternary assembly of the enzyme. As expected, the R28A mutant is drastically less thermostable than the WT enzyme (*T*_m_ ~ 53°C), while retaining a similar protection by nucleotides. The impact of the K108A mutation was less detrimental as its thermostability was moderately reduced (Δ*T*_m_ ~ 7°C), in agreement with its lower implication in the stabilization of the hexameric state.

**FIGURE 6:**
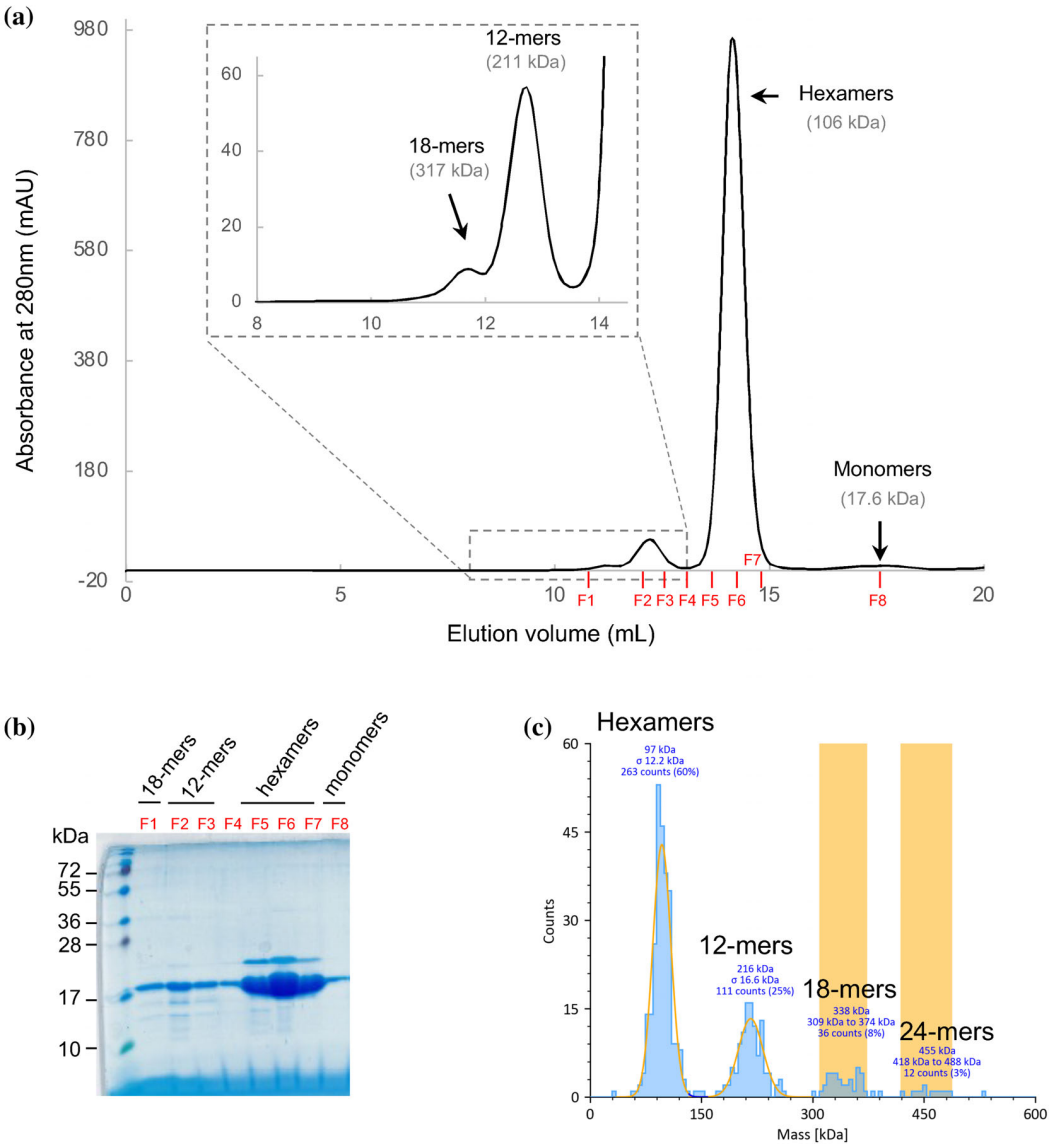
Size‐exclusion chromatography and mass photometry of *Streptococcus pneumoniae* nucleoside diphosphate kinase (SpNDK). (a) Size exclusion chromatography (SEC) profile for purified SpNDK. Elution peaks are shown with the estimated molecular weight of each different oligomerization state, deduced from a standard curve. The inset is a magnification of the two peaks corresponding to the 18‐ and 12‐mers. (b) The collected fractions (F1 to F8) as indicated in red in panel (a) were resolved on an 18% sodium dodecyl sulfate‐polyacrylamide gel electrophoresis gel. (c) Mass photometry profile for SpNDK with Gaussian peaks (high count numbers) and histograms (low count numbers) shown for the NDK different oligomeric states. The percentage of each oligomeric state is also indicated.

The purified WT SpNDK was further characterized by size‐exclusion chromatography and mass photometry (Figure 6). Both techniques confirmed that the predominant assembly of SpNDK is the hexameric state and a similar result was observed for the K108A mutant. In contrast, the R28A mutant eluted from a size‐exclusion chromatography essentially as lower molecular weight species (Figure S5), most likely a dimer, in agreement with its strongly reduced thermostability. For the WT SpNDK, however, these two approaches revealed unexpectedly that higher oligomeric species were also present in solution, either in concentrated (i.e., size‐exclusion, Figure 6a,b) or diluted (mass photometry, Figure 6c) samples, with the presence of 12‐, 18‐ and presumably 24‐Mers of SpNDK. Mutation of the catalytic Histidine into Alanine (H121A) seems to slightly reduce the formation of dodecamers or higher oligomeric species (Figure S6). Addition of ATP or GTP, on the other hand, reduce the formation of these supramolecular organizations in the WT enzyme but the most pronounced effect was observed with the H121A; this mutant did not form molecular species larger than the hexamer when ATP or GTP was added. In contrast, ADP or guanosine diPhosphate (GDP) did not have any significant effect on the WT enzyme suggesting that only the triphosphate nucleotide can modulate the higher oligomeric assemblies (Figure S6).

We also solved the cryo‐EM structure of hexameric SpNDK at a resolution of 2.47 Å, notably to confirm the presence of large oligomeric species. The map and model are presented in Figures S7 and S8 with data collection and refinement statistics in Table S2. As shown in Figure S8B, the model is highly similar to the crystal structure (rmsd of 0.364 over 125 C_α_). Interestingly, if the protein was mostly found in the hexameric state, we could isolate two similar dodecameric assemblies, refined to ~4 Å resolution (Figures S7 and S8). In both assemblies, the preferential orientations of the dodecamers in the frozen samples and the limited resolution of the maps (Figure S9) precluded atomic model refinement. Nonetheless, by fitting the cryo‐EM hexamers in both maps, we found that in each assembly, one NDK hexamer interacts with another one at an angle of 70–75°. Although identification of the precise interface between the two hexamers remains challenging, we tentatively proposed several residues—shown as sticks in Figure S8C—that could potentially stabilize the dodecameric form. However, we cannot rule out that the dodecameric state observed by cryo‐EM involves a different interface than that detected by SEC or mass spectrometry (Figure 6). Additional work will be required to elucidate precisely how the two hexamers, and higher oligomeric states, interact.

## 3 DISCUSSION

In this study, we solved the crystal structure of SpNDK in both the apo state and the ADP‐Vi‐bound state, revealing that the enzyme adopts a canonical hexameric conformation. In many NDKs, this hexameric state is often stabilized by a C‐terminal extension that supports the trimer‐of‐dimers arrangement (Dumais et al., 2018) (Figures S2 and S3). This extension has also been reported to influence the hexamer stability in human NDKs (Lim & Natarajan, 2024), and in the *Dictyostelium discoideum* enzyme when its deletion is combined with a P100S mutation (“Killer of prune” mutation) (Karlsson et al., 1996). The C‐terminus of SpNDK is, however, relatively short, a feature shared with some bacterial NDKs that forms exclusively tetramers (Georgescauld et al., 2020). Therefore, the hexameric assembly of SpNDK must be stabilized by other regions of the protein. Here, hexamer integrity is notably secured by the *Kpn*‐loop, as well as by residues located between β‐strands 1 and 2. In *M. tuberculosis*, the NDK enzyme (MtNDK) (Chen et al., 2002) also lacks a C‐terminus extension, yet it forms hexamers that are very stable with a melting temperature of ~76°C (Chen et al., 2002), similar to what we found for SpNDK. In *M. tuberculosis*, this thermal stability has been attributed to the presence of a salt bridge formed between Arg80 of one subunit and Asp93 of another subunit from a neighboring dimer (Georgescauld et al., 2013). Disruption of this salt bridge by the mutation of Asp93 into asparagine prevented the formation of six D93‐R80 salt bridges in the whole hexamer, thereby leading to a dramatic *T*_m_ decrease of ~28°C (Georgescauld et al., 2013). Arginine residues are frequently found at the subunit–subunit interface (Hu et al., 2000; Moreira et al., 2007), and their ability to create up to five H‐bonds with their side chain—in particular with carboxyl groups of the main chain residues—is an efficient strategy used by proteins throughout evolution to increase their stability (Borders Jr. et al., 1994). In *S. pneumoniae*, the stabilization of the trimer interface within the hexamer involves two arginine residues, Arg15 and Arg28, from one subunit, each forming H‐bonds with residues from a neighboring dimer. Arg28 especially contributes to the stabilization of the trimer by forming four H‐bonds with two main chain carboxyl groups, including Pro94 in the *Kpn*‐loop from a neighboring subunit. Consequently, substitution of Arg28 by an alanine residue has a dramatic effect on the thermostability of SpNDK (∆*T*_m_ ~ 23°C) as well as on the oligomeric state of the protein, leading to a complete destabilization of the hexameric form. As previously noted, the presence of an arginine residue at this position in NDKs is quite rare in the bacterial kingdom and appears to be the main driver for the hexamer stability. Interestingly, the sequences of NDKs from two different halobacteria, *Haloarcula quadrata* and *Haloarcula sinaiiensis*, differ only by a single residue, Arg31 and Cys31, respectively (Yamamura et al., 2009), equivalent to Arg28 in SpNDK. *H. quadrata* NDK is quite stable as a hexamer over a wide range of salt concentrations whereas *H. sinaiiensis* NDK dissociates into dimers at salt concentrations below 1M. The structure of the former enzyme was solved showing that Arg31 forms two hydrogen bonds with the main chain oxygen of Pro96 and Gly109 in the *Kpn*‐loop (Yamamura et al., 2009). In Drosophila, the mutation of the residue equivalent to Pro94 into Ser (“Killer of prune” mutation) has been shown to cause a substantial reduction in protein thermostability (Timmons et al., 1995), with the melting temperature decreasing from 71°C for the WT protein to 57°C for the mutant (Lascu et al., 1992), while the enzyme activity was unaffected. Another residue from the *Kpn*‐loop, Lys108, participates also in hexamer stability but to a much lower extent as its mutation into an alanine moderately affects the thermostability of the enzyme, while maintaining the hexamer integrity. Despite the large variability in the *Kpn*‐loop sequences and in subunit–subunit interactions derived thereof, these results underscore the key role of this region of the protein in the stabilization of the quaternary structure of many hexameric NDKs, and how variations across species reflect evolutionary adaptations linked to distinct functional constraints (Yamamura et al., 2009). SpNDK is a vivid example of this property, with both conserved and unique features in the residues mediating subunit interactions among bacterial enzymes.

The high thermostability of SpNDK was unexpected as this property is generally characteristic of thermophilic bacteria (Akanuma et al., 2013; Garcia et al., 2017). For example, the NDKs of two mesophilic bacteria, *E. coli* and *Bacillus subtilis*, have a melting temperature below 60°C, whereas the NDK of *Vibrio cholerae* exhibits an even lower *T*_m_ (44°C) (Agnihotri et al., 2021). In contrast, the NDK from the thermophilic archaeon *Methanothermobacter thermautotrophicus* has a *T*_m_ of approximately 80°C (Akanuma et al., 2013). Because NDKs from pathogenic micro‐organisms are often secreted and act as virulent factors (Yu et al., 2017), they must withstand various harsh environments within host cells (Souza et al., 2011). Consequently, it has been proposed that the high stability of some NDKs is an intrinsic property that enables the enzyme to maintain catalytic activity under hostile conditions (Georgescauld et al., 2020; Souza et al., 2011; Vieira et al., 2015). *S. pneumoniae* can thrive in multiple human organs, and its spreading is notably controlled by macrophages (Narciso et al., 2025). Although it is currently unknown whether SpNDK can be secreted by *S. pneumoniae*, this bacterium can undergo spontaneous autolysis, releasing its intracellular content into the surrounding milieu (Lewis, 2000). This mechanism is notably responsible for the release of pneumolysin, one of the major virulence factors of *S. pneumoniae* (Paton, 1996), into infected tissues (Martner et al., 2008). Therefore, it is tempting to speculate that the high stability of SpNDK could be advantageous after autolysis, enabling the enzyme to resist host defenses and potentially promoting *S. pneumoniae* infection.

The oligomeric state, in particular for NDKs that assemble into hexamers, is required for the NDK activity (Georgescauld et al., 2013). This is because it stabilizes the *Kpn*‐loop at the interface of the trimers‐of‐dimers assembly (Dautant et al., 2017), a loop that is directly involved in nucleotide binding (Georgescauld et al., 2020). Beyond the canonical hexameric state, a key finding from our study is the ability of SpNDK to form supramolecular structures, suggesting a potential new layer of regulation for the diverse cellular functions of NDKs. Indeed, it is well‐known that variation in oligomerization status provides an efficient means to diversify the protein functions within the cell (Jeffery, 1999). For example, the B‐isoform of human NDK typically assembles as hexamers (Gilles et al., 1991). However, its ability to bind single‐strand DNA (Hildebrandt et al., 1995) has been shown to be linked to the dimeric form of the enzyme (Mesnildrey et al., 1997). Similarly, purified NDKs from *E. coli* or *V. cholerae* exist in equilibrium between a canonical tetramer and a dimer (Agnihotri et al., 2021; Shen et al., 2006). This equilibrium occurs at physiologically relevant concentrations for *E. coli* enzyme (Shen et al., 2006), supporting the possibility of a yet uncharacterized functional role for the dimer. Nucleotide binding shifts this equilibrium in vitro: ATP or ADP promotes tetramer formation, indicating that the concentration of nucleotides is a key regulator of the protein oligomeric state (Shen et al., 2006).

Finally, some studies have briefly reported the occurrence of NDK dodecamers in plants (Huang et al., 2005; Kopylov et al., 2015), with one case demonstrating functional activity of this form using blue native gels (Kopylov et al., 2015). Although these observations were not further discussed in these studies, they hint that supramolecular assemblies may also occur in other NDK species. Moreover, the formation of NDK filaments, or rod‐like polymers, have been described in human NDK isoform B (Morin‐Leisk & Lee, 2008), and for NDK from *Trypanosoma cruzi* parasite (Gomez Barroso et al., 2022). The former, which are few μm in length, formed exclusively in the presence of nucleotide, with GTP being the most efficient. These NDK polymers did not form when the catalytic histidine residue was mutated and required high enzyme concentrations (>5 μM) (Morin‐Leisk & Lee, 2008). *T. cruzi* NDK filaments, on the other hand, disassembled rapidly in the presence of ATP or GTP (Gomez Barroso et al., 2022). In this regard, our findings on SpNDK appear somehow related to those of the parasitic enzyme. Importantly, many enzymes involved in intermediary metabolism have been shown to form filaments (Hvorecny & Kollman, 2023). Yet, the interfaces between the subunits that form these supramolecular structures are not necessarily conserved across the species and might have been tailored differentially throughout evolution with polymerization occurring multiple times (Hvorecny & Kollman, 2023).

In conclusion, our work establishes the structural basis of SpNDK oligomerization at atomic resolution and reveals unprecedented supramolecular assemblies for a bacterial enzyme. These assemblies could potentially modulate the enzymatic activity or interactions with cellular partners, given the remarkable functional diversity of NDKs and their involvement in various cellular processes. Further studies will determine whether this novel property is conserved across other bacterial NDKs and how it may impact their physiological roles.

## 4 MATERIALS AND METHODS

### 4.1 Plasmid construction

NDK carrying a N‐terminal hexa‐histidine tag (His‐SpNDK) was amplified by polymerase chain reaction (PCR) from *S. pneumoniae* genomic DNA using the forward CATATGGAACAAACATTCTTTATCATCAAACCA and reverse primer GGATCCTTAAAACCAAAGAGCAATTTC TCG, containing, respectively, a *Nde*I or *Bam*HI restriction site. The *Nde*I/*Bam*HI digested fragment was ligated into pET28 pre‐digested with the same enzymes. The final expression plasmid resulted in pET28‐*his*‐*ndk* which encodes a 157 amino acid polypeptide including an N‐terminal histidine tag, referred to as His‐SpNDK in the text.

The same cloning strategy was used to construct SpNDK‐His carrying a C‐terminal hexa‐histidine tag, except that the primers for the PCR were 5′‐CGATACCATGGAACAAACATTCTTTATCATCA AACCA‐3′, and CGTTACTCGAGAAACCAAAGAGCAATTTCTCG‐3′, containing, respectively, a *Nco*I and *Xho*I restriction site. *E. coli* BL21(DE3) cells were transformed with the plasmid encoding either His‐SpNDK or SpNDK‐His.

### 4.2 Site‐directed mutagenesis

The R28A, K108A, and H121A mutations were engineered in His‐SpNDK using the QuikChange Lightning Site‐Directed Mutagenesis Kit (Agilent Technologies) according to the manufacturer's instructions. The following primers were used for the mutagenesis of the R28A: GTGAAGTGTTAAAGCGCATCGAACAAGCTGGATTTACAATCGAAAAA and TTTTTCGATTGTAAATCCAGCTTGTTCGATGCGCTTTAACACTTCAC. For the H121A mutant, the primers were: CCAAAATGTTGTAGCGGGTTCAGATTCAG and CTGAATCTGAACCCGCTACAACATTTTGG. For the K108A, the primers were: AGGCACTATTCGAGGTGATTTTGCAGCAGCTGCTGGAGAA and TTCTCCAGCAGCTGCTGCAAAATCACCTCGAATAGTGCCT. DNA sequences were verified by Sanger sequencing (microsynth). The underlined letters indicate the restriction enzyme cleavage sites.

### 4.3 Protein purification

For the WT SpNDK‐His, the NDK protein carrying a C‐terminal hexa‐histidine tag was produced in *E. coli* BL21(DE3) transformed with pET28‐*ndk*‐his. Bacteria were grown at 37°C in lysogeny broth (LB) media supplemented with 50 μg.mL^−1^ kanamycin. Protein expression was induced at a DO_600_ of 0.5 by the addition of 1 mM isopropyl β‐D‐1‐thiogalactopyranosid (IPTG). After 16 h of incubation at 18°C, bacteria were harvested by centrifugation at 6000 × *g* at 4°C for 15 min and resuspended in lysis buffer (100 mM 4‐(2‐HydroxyEthyl)‐1‐PiperazinEthaneSulfonic acid (HEPES) pH 7.4, 150 mM NaCl and 5% (w/v) glycerol). Cells were lysed by sonication with 10 cycles of 30 s with 30 s interval between each pulse and the lysate was centrifuged at 10,000 × *g* for 30 min at 4°C. The supernatant containing spNDK His was loaded onto a Nickel affinity column (HisTrap™ Fast Flow, Cytiva) equilibrated with lysis buffer containing 40 mM imidazole. Bound proteins were eluted in lysis buffer supplemented with 400 mM imidazole. The eluate containing spNDK‐His was loaded onto a size‐exclusion chromatography column (Superdex™ 200 Increase 10/300 GL, Cytiva) equilibrated with 50 mM HEPES pH 7.4, 50 mM NaCl and 1 mM ethylenediaminetetraacetic acid. Fractions of interest were pooled and concentrated to 10 mg.mL^−1^ using an Amicon® Ultra‐4 centrifugal device (Millipore®) with a molecular weight cut‐off of 3 kDa.

For WT His‐SpNDK, the NDK protein carrying a N‐terminal hexa‐histidine tag was produced in *E. coli* BL21(DE3) transformed with pET28‐*his‐ndk*. Bacteria were grown at 37°C in 500 mL of LB media supplemented with 50 μg.mL^−1^ kanamycin for 4–6 h until a DO_600_ of 0.6 was reached. The culture was then induced with 0.7 mM IPTG for 1 h at 25°C, and centrifuged at 7000 × *g* for 10 min at 4°C. The bacteria were washed with 35 mL of buffer 50 mM Tris pH 8.0 and 5 mM MgCl_2_, and finally resuspended in 50 mM Tris pH 8.0, 5 mM MgCl_2_, 1 mM diThioThreitol, protease inhibitor cocktail (0.1% chymostatin, 0.1% pepstatin, 0.1% leupeptin, 0.1% antipain, and 0.4% aprotinin) and 1 mM DNase. The cells were lysed by sonication with 6 cycles of 20 s and 20 s of interval between each impulsion. The lysate was then centrifuged at 150,000 × g for 1 h at 4°C and 2 mL of Nickel‐NitriloTriAcetic acid resin previously equilibrated with 50 mM HEPES pH 7.4, 100 mM NaCl and 10 mM imidazole were added to the supernatant. The resin was then washed 5 times with 3 mL of the same buffer, and bound proteins were eluted with twice 2 mL of buffer supplemented with 300 mM imidazole. The proteins were centrifuged at 15,000 × g for 15 min at 4°C to remove the aggregates before injection onto a Superdex 200 Increase 10/300 GL column, previously equilibrated with 50 mM HEPES pH 7.4 and 100 mM NaCl. The R28A, K108A, and H121A his‐SpNDK mutants were purified according to the same protocol as the WT enzyme.

### 4.4 Protein crystallization and structure determination of the NDK structures

His‐SpNDK was crystallized at a concentration of 17.5 mg.mL^−1^ by mixing 0.5 μL with an equal volume of precipitation reagent in an MRC 2‐drop plate (Molecular Dimensions), using the liquid‐handling robot Mosquito (TTP). The reservoir volume was 70 μL. Crystals of the apo protein were obtained at 20°C in 0.2 M calcium chloride dihydrate, 0.1 M HEPES sodium pH 7.5 and 28% (v/v) polyEthylene glycol (PEG) 400. Crystals were directly flash‐frozen in liquid nitrogen and diffracted remotely on beamline ID30A‐3 (MASSIF‐3) at the European Synchrotron Radiation Facility (ESRF) synchrotron. For the ADP‐bound structure, SpNDK‐His was used. Crystals were obtained by mixing an equal volume of protein at 18 mg.mL^−1^ and reservoir solution composed of 24% (v/v) PEG 3350, 100 mM 2‐(N‐Morpholino)EthaneSulfonic acid (MES) pH 7.0 in a final volume of 1.7 μL. Crystallization was performed at 20°C by the hanging drop vapor diffusion method in 24‐well XRL plates (Molecular Dimensions), over a reservoir solution of 500 μL. Ligands were soaked into the crystal‐containing drop by adding a solution of orthovanadate (5 mM final) previously heated at 90°C for 5 min and MgADP (5 mM final). After 5 min, crystals were cryoprotected with 15% of ethylene glycol before being flash‐frozen in liquid nitrogen and remote diffraction on beamline ID23‐2 (ESRF).

For both datasets, images were indexed and integrated with iMosflm, then scaled and merged with Aimless (Evans & Murshudov, 2013). The apo structure was solved by molecular replacement using Phaser (McCoy et al., 2007) with the NDK model from *S. aureus* (Protein Data Bank [PDB] 3Q8Y) as the search probe, after removal of ligands. The apo model was then used to solve the liganded‐NDK structure. The unit cell contains one monomer per asymmetric unit for the apo structure and six for the ADP‐vanadate structure. The models were refined with cycles of restrained refinement with REFMAC5 (Murshudov et al., 2011) and manual building in Coot (Emsley et al., 2010). The final structures were validated using Procheck (Laskowski et al., 1993) and Rampage (Lovell et al., 2003). Data collection and refinement statistics are listed in Table S2.

### 4.5 Nano differential scanning fluorimetry

The nanoDSF experiment was performed using a Prometheus NT.48 instrument (NanoTemper Technologies) in a buffer composed of 50 mM HEPES pH 7.4 and 50 mM NaCl. When specified, ATP or GTP was added at a final concentration of 4 mM in the presence of 5 mM MgCl_2_. Proteins were loaded into nanoDSF grade standard capillaries (NanoTemper Technologies) and exposed to a thermal gradient ranging from 20 to 95°C with a temperature increase of 1°C every minute. Samples were excited at 280 nm, and the emission of the tryptophan and tyrosine fluorescence was measured at 330 and 350 nm using a dual‐UV detector. Data were analyzed using PR stability analysis software (NanoTemper Technologies).

### 4.6 Mass determination by mass photometry

All measurements were acquired using a commercial Refeyn OneMP mass photometer. The buffer solution (50 mM HEPES pH 7.4, and 100 mM NaCl) was filtered onto an Amicon Ultra‐4 spin centrifugal filter device with a molecular weight cut‐off of 30 kDa (Millipore®). The standard native marker (NativeMark™ LC0725, ThermoFisher Scientific) was used for calibration applying three reference points with a maximum calibration error of 5%. The His‐SpNDK protein was diluted to 200 nM in filtered buffer. For analysis, 15 μL of filtered buffer was added to an empty well on the cover slide, along with 5 μL of the pre‐diluted protein solution. Measurements were acquired using the Acquire MP v2.5.0 program and ratiometric images (data) were analyzed using the Refeyn Discover MP software (Refeyn Ltd.). For the effect of nucleotides, the buffer solution (50 mM HEPES pH 7.4, and 150 mM NaCl) was supplemented with either ATP, GTP, ADP, or GDP in the presence of 5 mM MgCl_2_, and the proteins were incubated for 5 min at 22°C before the analysis was performed.

### 4.7 Cryo‐EM sample preparation and data collection

A volume of 3 μL of purified His‐spNDK at 0.25 mg.mL^−1^ was applied onto a Quantifoil Au‐200‐mesh R1.2/1.3 grid previously glow discharged with ELMO™ system (Cordouan Technologies) for 40 s with 2.7 mA current. Excess solution was blotted for 4 s and vitrification was performed in liquid ethane using a FEI Vitrobot Mark IV (Thermo Fischer Scientific) at 4°C and 100% humidity.

The grid was imaged using a Glacios TEM (Thermo Fischer Scientific) equipped with a Falcon 4i direct electron detector and a Selectris X energy filter. The sample was imaged at a nominal magnification of 165,000× corresponding to a calibrated pixel size of 0.694 Å. A total of 3184 movie frames with an electron dose of 59.5 e^−^/Å^2^ over 42 frames were collected using EPU software (Thermofisher Scientific) with a defocus range of −0.5 to −1.7 μm (steps of −0.2 μm).

### 4.8 Cryo‐EM image processing

Data analysis was carried out using cryoSPARC v4.6.0 (Punjani et al., 2017). Movies were corrected for gain reference, motion‐corrected using Patch motion correction and the contrast transfer function (CTF) was determined using Patch CTF. For NDK hexamer, a total of 3,231,210 particles were picked using blob picker and subsequently extracted. After two rounds of 2D classification to remove 2D class averages with less resolved features or presenting particles in close contact, the selected 716,048 particles were used to build five ab initio maps. The best isotropic map was used as the initial reference to calculate a new map from the 716,048‐particle dataset using hetero‐refinement classification. The hexamer map was further improved using non‐uniform refinement by imposing D3 symmetry, yielding a map with resolution at 2.47 Å. For NDK dodecamer, a total of 1,653,163 particles were picked using automated blob picker with an elliptical shaped blob. After two rounds of 2D classification to remove 2D class averages with less resolved features or presenting a single hexamer, the selected 148,143 particles were used to generate three ab initio maps. Two maps were obtained with a dodecamer organization. The corresponding particles were separated into five 3D classes using 3D classification leading to two alternate conformations. The first and second maps (dodecamer 1 and 2), comprising 62,236 and 51,255 particles, respectively, were refined independently using non‐uniform refinement without imposing symmetry. The final resolutions of dodecamer 1 and 2 were 4.09 and 3.81 Å, respectively.

### 4.9 Cryo‐EM model building

A first monomer model was generated using the “Predict and Build” tool implemented in Phenix (Liebschner et al., 2019) from the cryo‐EM hexamer data. This model was refined using Isolde (Croll, 2018) and Phenix. Structure validation was performed with the MolProbity program (Chen et al., 2010; Williams et al., 2018). Data collection and refinement statistics are listed in Table S2.

## AUTHOR CONTRIBUTIONS

**Laetitia Daury:** Funding acquisition; project administration; supervision; writing – review and editing. **Jean‐Michel Jault:** Conceptualization; funding acquisition; project administration; supervision; validation; visualization; writing – original draft; writing – review and editing. **Olivier Lambert:** Conceptualization; data curation; formal analysis; project administration; supervision; validation; visualization; writing – review and editing. **Paul Nouri:** Investigation; formal analysis; writing – review and editing. **Elise Kaplan:** Conceptualization; data curation; formal analysis; investigation; project administration; supervision; validation; visualization; writing – review and editing. **Marie‐France Giraud:** Formal analysis; validation; writing – review and editing. **Lionel Ballut:** Data curation; formal analysis; investigation; validation; writing – review and editing. **Julie Kerboeuf:** Formal analysis; investigation; writing – review and editing. **Cédric Orelle:** Supervision; writing – review and editing. **Cécile Gonzalez:** Conceptualization; formal analysis; investigation; project administration; supervision; validation; visualization; writing – review and editing. **Frédéric Galisson:** Formal analysis; investigation; validation; writing – review and editing.

## FUNDING INFORMATION

This study was funded by the ANR (“Agence Nationale de la Recherche,”) grant N° 22‐CE11‐0018 to Jean‐Michel Jault and Olivier Lambert.

## CONFLICT OF INTEREST STATEMENT

The authors declare that they have no conflicts of interest related to this research.

## Supporting information


**Data S1.** Supporting Information.

**Data S2.** Supporting Information.

**Movie S1.** Tour of the electron density surrounding NDK in complex with ADP and vanadate. The movie starts with an overview of the protein structure and then sequentially zooms into representative secondary‐structure elements to illustrate the quality of the 2F_o_ − F_c_ electron density map (contoured at 1*σ*) and its agreement with the refined atomic model. The camera pauses at the regions highlighting α‐helices, β‐sheets, and loop regions with side chains displayed as sticks. The movie concludes with a close‐up view of the electron density surrounding the bound ADP molecules in the nucleotide‐binding site.

## Data Availability

The coordinates and structure factors for the NDK crystal models have been deposited in the Protein Data Bank (PDB) under accession numbers 9RVW (apo form) and 9SFO (ADP‐Vi complex). The cryo‐EM atomic model and maps have been deposited in the PDB and Electron Microscopy Data Bank (EMDB) under the accession codes 9SLP and EMD‐55011 for the NDK hexamer, and EMD‐55334 and EMD‐55333 for the NDK dodecamer 1 and 2 maps, respectively.
