# Supplementary material for: Quaternary structures of Streptococcus pneumoniae nucleoside diphosphate kinase: From hexamers to supramolecular assemblies
Source: Protein Sci. 2026 Aug 8;35(9):e70735. doi: 10.1002/pro.70735 (PMC13451967; doi:10.1002/pro.70735)
Supplement: Supplementary file 1 [file PRO-35-e70735-s002.pdf]

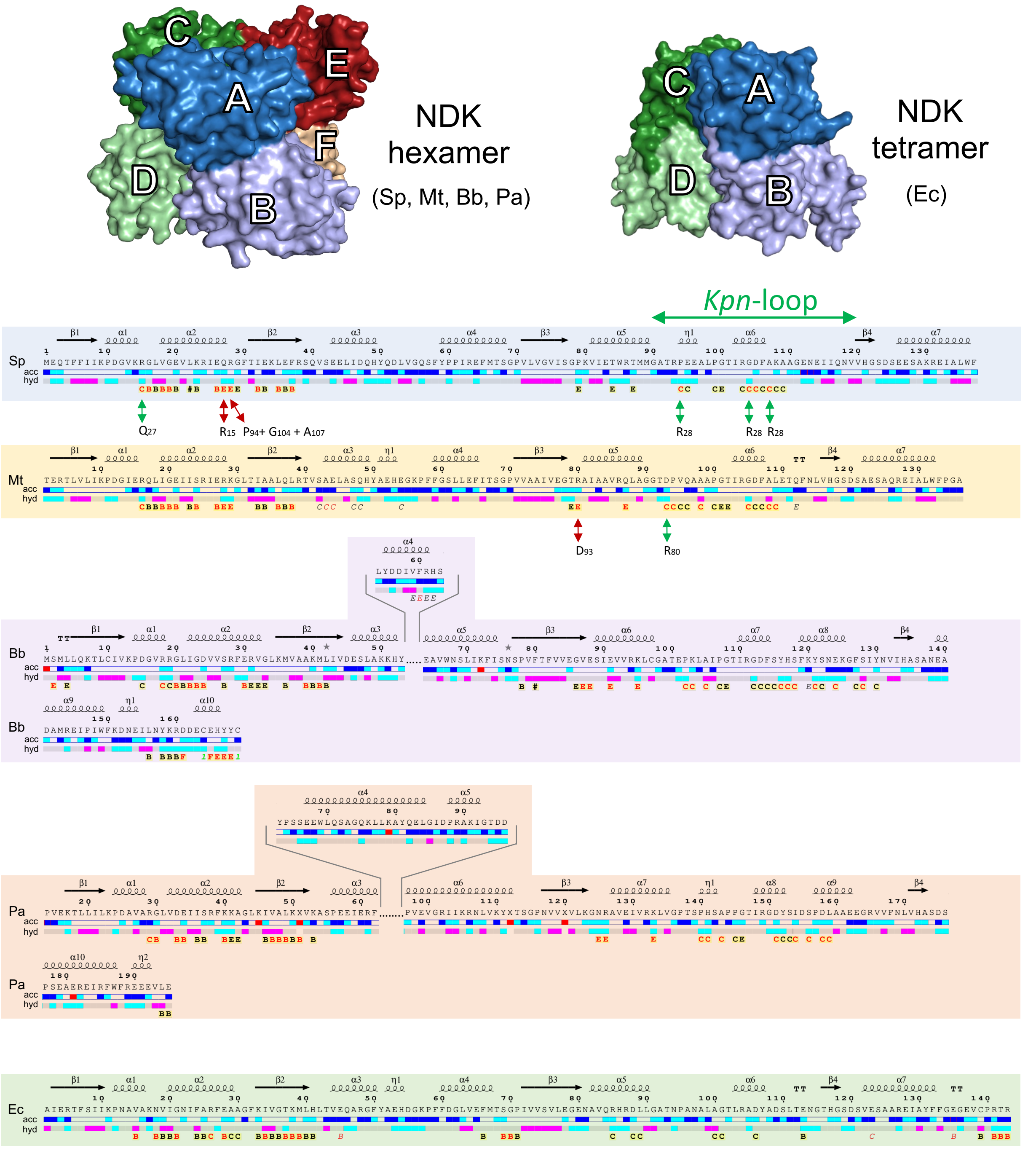

Figure S1. Secondary structures and intermolecular contacts for selected NDKs. The secondary structures are shown above the sequences and were derived from the 3D structures of SpNDK (PDB 9RVW), *Mycobacterium tuberculosis* (1K44), *Borrelia burgdorferi* (4DI6), *Pyrobaculum aerophilum* (1XQI) and *Escherichia coli* (2HUR). The four first NDKs assemble as hexamers (top left panel) and the last NDK, from *E. coli*, forms tetramers (top right panel), with the subunit arrangement indicated. Residues adopting alternative conformations are highlighted by a grey star above the sequence. The accessibility (acc), hydropathy scales (hyd), and intermolecular contacts (bottom lines underneath the sequences) are shown. The intermolecular contacts between the A subunit (query) and the other subunits from the biological assembly (e.g. subunits C, B and E for SpNDK) are indicated. For intermolecular contacts < 3.2 Å, the letter is in red, and for contacts between 3.2-5.0 Å, the letter is in black. For SpNDK and *M. tuberculosis* NDK, the residues involved in the main interactions are indicated by double-sided arrows (e.g. between R_{15} in the A-subunit of SpNDK and Q_{27} in the C-subunit) with interactions between A- and C-subunits, and A- and E-subunits, in green and firebrick colours, respectively. Green numbers 1 below the *B. burgdorferi* sequence indicate the formation of a disulfide bond. The figure was drawn with ENDscript 2.0 [58].

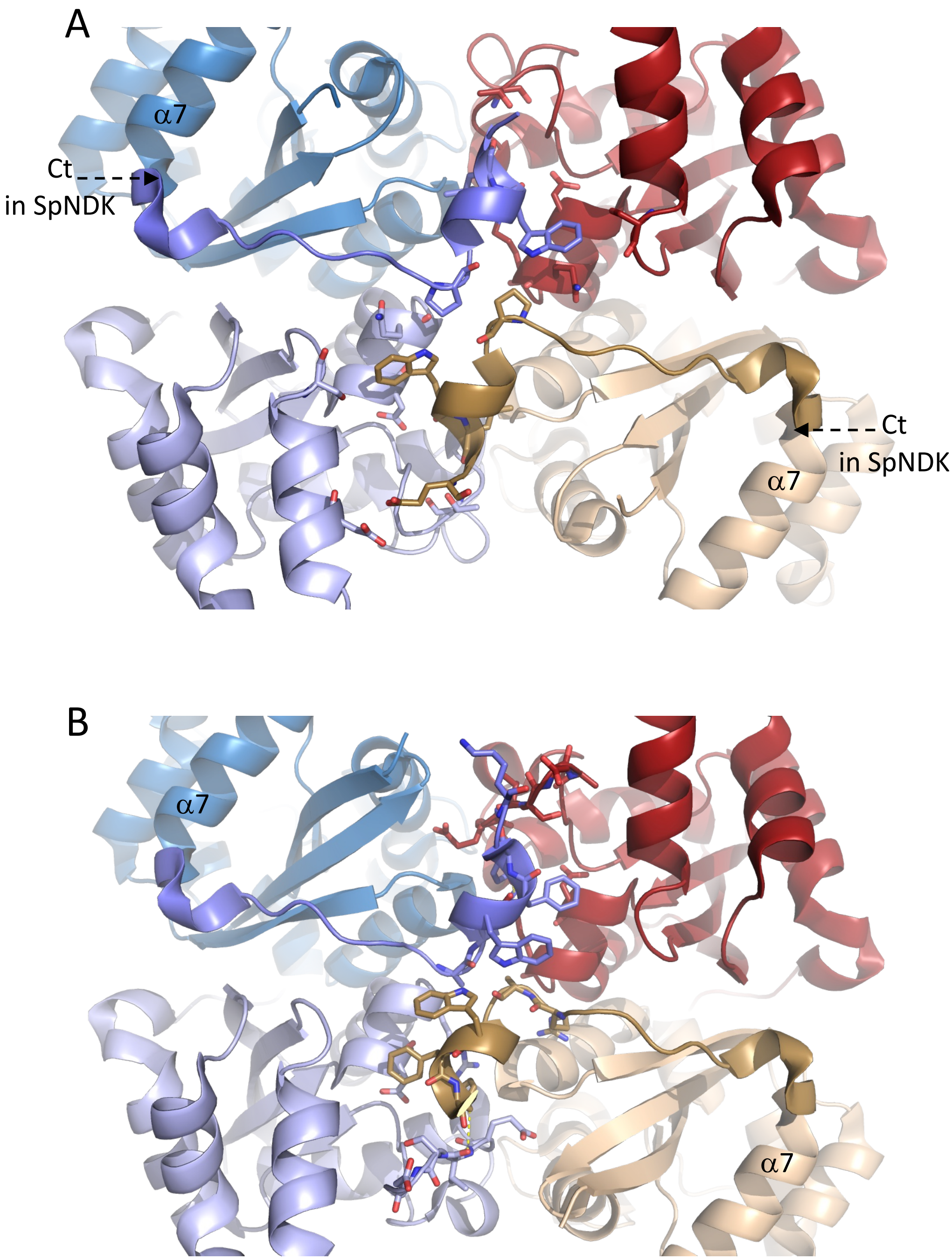

Figure S2. Role of the C-terminus extension in the hexamer stabilization of some NDKs. Network of interactions provided by the C-terminus extension in the NDKs from *S. aureus* (3Q83 ; panel **A**) or *Pyrococcus horikoshii* (2CWK ; panel **B**). The subunits are colored according to Fig. S1: sky-blue for the A subunit with the C-terminus extension colored in slate, light blue for the B subunit, firebrick for the E subunit, and wheat for the F subunit with the C-terminus extension colored in sand. The residues involved in interactions ($d \leq 4.0$ Å) between the C-terminus extension of the A- or F-subunits and the E-/F-subunits or the A-/B-subunits (i.e. from the neighboring dimers), respectively, are shown as sticks. Please note that there is no C-terminus extension in the SpNDK structure (see Figure 2), which ends right at the end of the α-helix 7 (indicated by a dashed arrow in panel A).

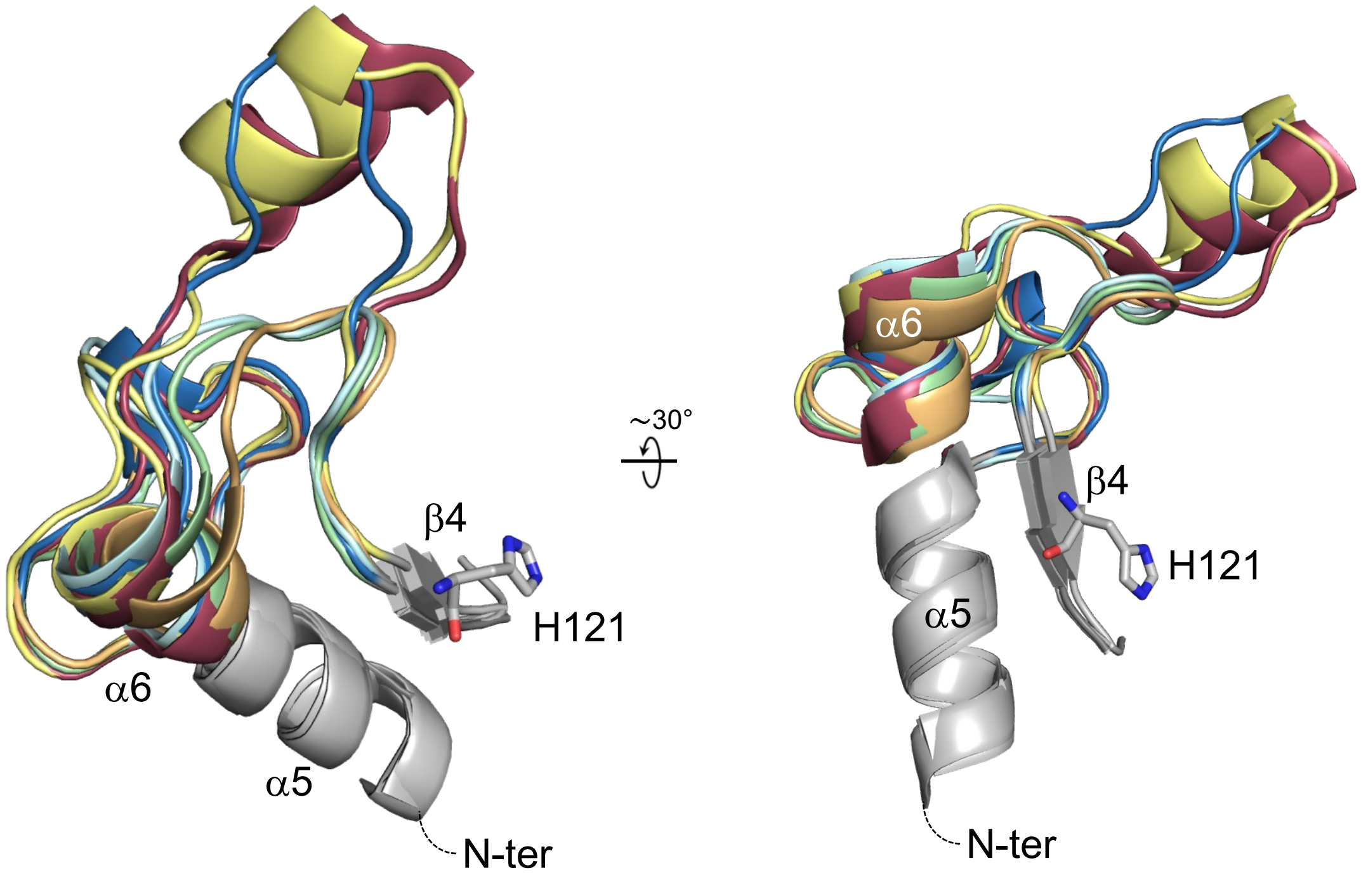

Figure S3. Structural variations in the second part of the *Kpn*-loops of NDKs. The *Kpn*-loops, located between the α-helix 5 and the β-strand 4 (in grey colors) and using the SpNDK as a reference (see Figure 2 for the secondary structure numbering), are colored as follows: SpNDK (9RVW), sky-blue ; *Mycobacterium tuberculosis* NDK (1K44), light blue ; *Staphylococcus aureus* NDK (3Q8Y), pale green; *E. coli* NDK (2HUR), light orange ; *Pyrobaculum aerophilum* NDK (1XQI), pale yellow ; *Borrelia burgdorferi* (4DI6), raspberry. The catalytic histidine of SpNDK (H121) is shown as sticks. In the first part of the *Kpn*-loop, until the end of α-helix 6, all NDKs adopt a similar folding but the second part of the loops diverge both in sequence and in length.

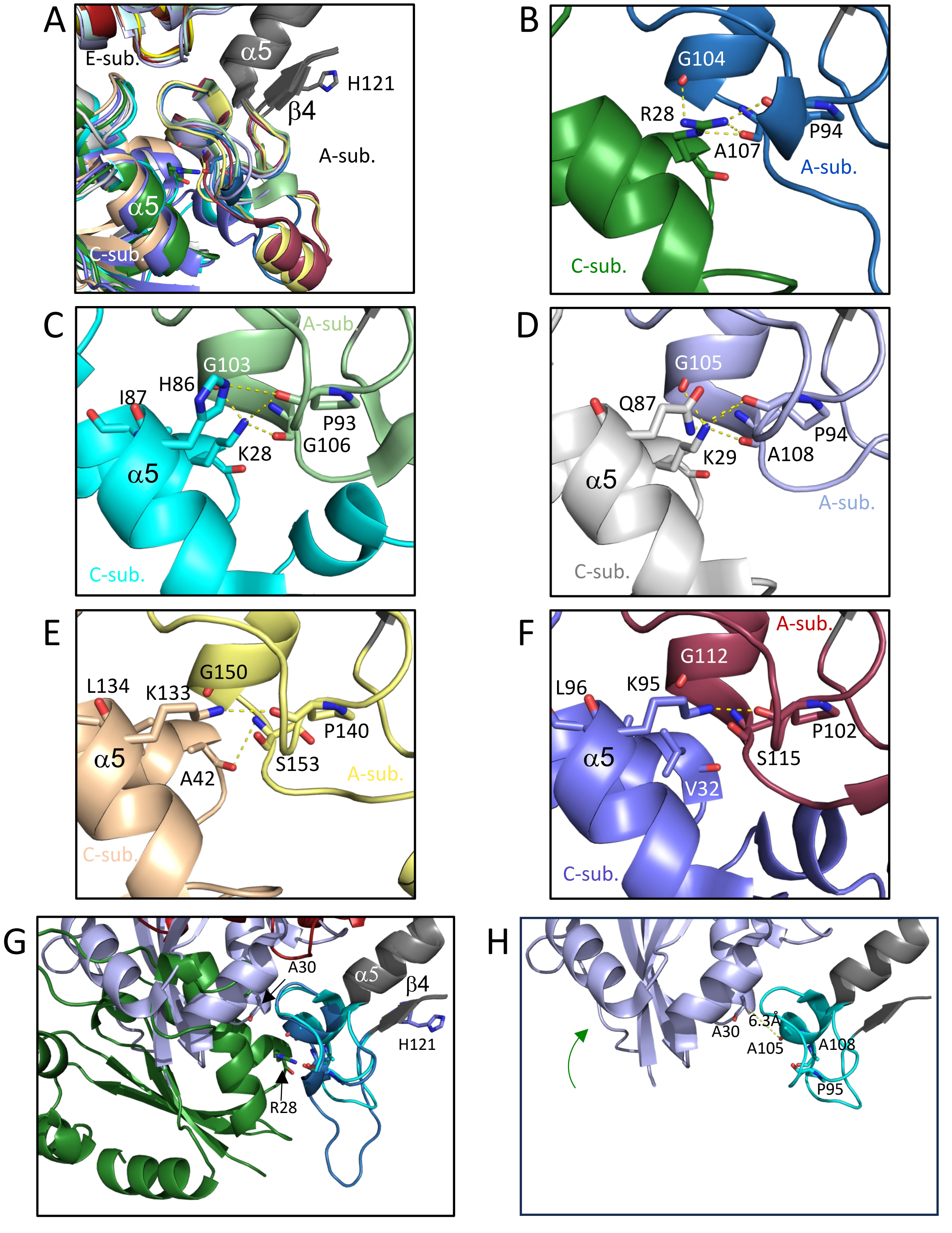

Figure S4. Conservation of three 'connecting residues' in the first part of the *Kpn*-loops, both in hexameric and tetrameric NDKs. (see legend next page)

Figure S4. Conservation of three 'connecting residues' in the first part of the *Kpn*-loops, both in hexameric and tetrameric NDKs. A, overlay of different hexameric NDKs (see below) on the A-subunit SpNDK (apo form). For the A-subunits, only the *Kpn*-loops are displayed and the α-helix 5 and β-strand 4 are colored in gray. **B** to **F**, zoom on the interface between the three connecting residues from the *Kpn*-loop of the A-subunit and the C-subunit. **B**, SpNDK ; **C**, *Staphylococcus aureus* NDK (3Q83); **D**, *Mycobacterium tuberculosis* NDK (1K44) ; **E**, *Pyrobaculum aerophilum* NDK (1XQI) ; **F**, *Borrelia burgdorferi* NDK (4DI6); **G**, Overlay on the A-subunit of the hexameric SpNDK (apo form) with the tetrameric *E. coli* NDK. The color coding is the same as in Figure 1 for SpNDK and only the A-, C- and E-subunits are shown from the top of the hexamer. For *E. coli* NDK, only the A- and C-subunit (colored cyan and pale blue, respectively) are shown. For SpNDK, H121 and the P94, G104, A107, from the A-subunit and R28 from the C-subunit are shown as sticks. **H**, the equivalent connecting residues residues in the A-subunit of *E. coli* NDK are also shown as sticks, namely P95, A105, A108, and the A30 in the C-subunit is shown as well. The C-subunit in *E. coli* NDK tetramer is rotated in a clockwise direction as compared to the C-subunit in the hexameric SpNDK, as indicated by the green arrow. This leads to a higher distance between A30 from the C-subunit, and P95, A105, A108, from the A-subunit.

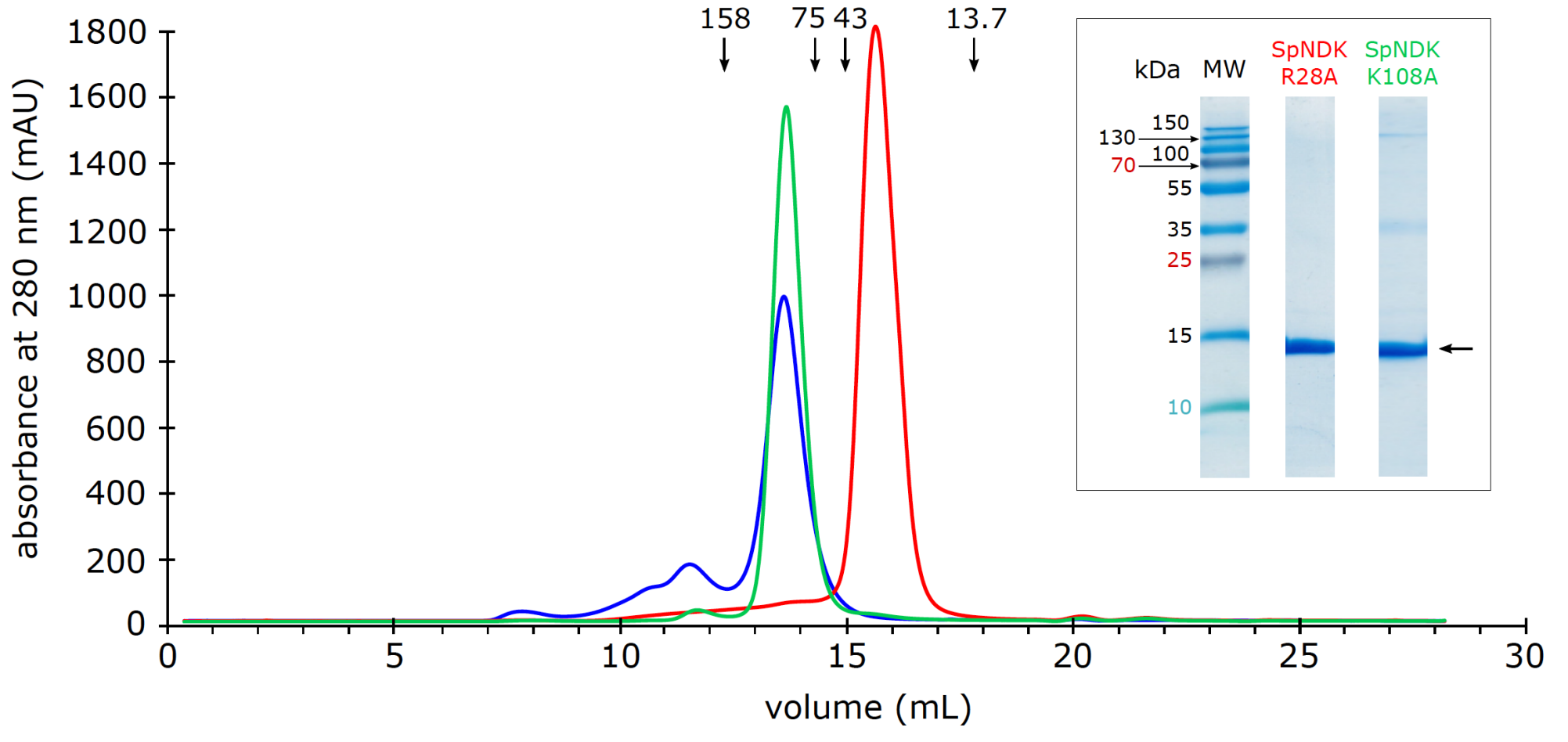

Figure S5. Size-exclusion chromatography and SDS-PAGE of the R28A and K108A mutants. The SEC profile is shown in red for the R28A mutant, in green for the K108A mutant and in blue for the WT SpNDK as comparison. The sizes of the molecular weight markers, expressed in kDa, are shown above the SEC profiles and correspond to aldolase (158 kDa), conalbumin (75 kDa), ovalbumin (43 kDa) and ribonuclease A (13.7 kDa). The elution volume of the R28A mutant corresponds to a molecular weight of ~ 34.3 kDa, as estimated from a standard curve made from the molecular weight markers. The inset shows the SDS-PAGE of the purified R28A and K108A mutants with the arrow on the right corresponding to the SpNDK protein.

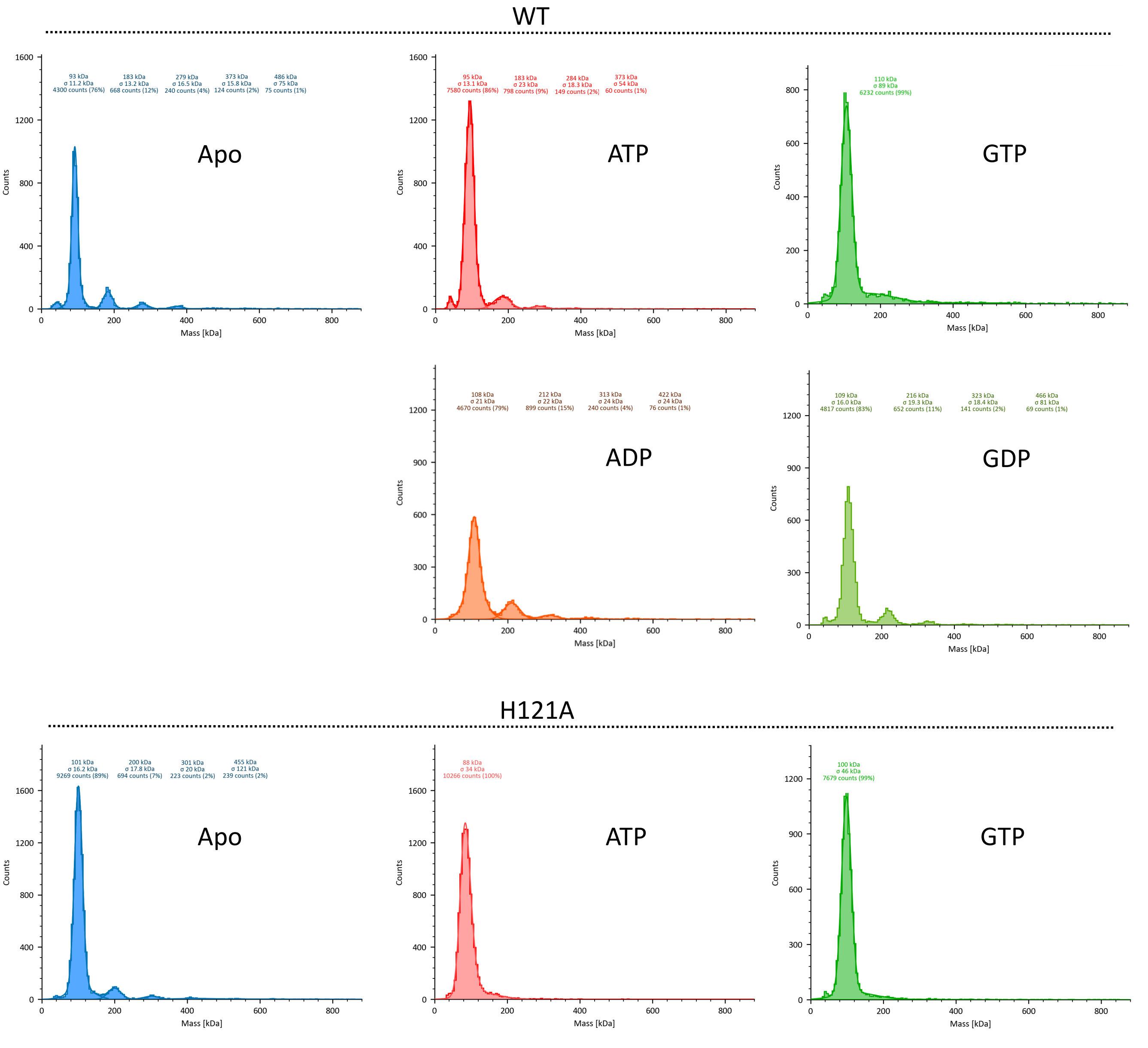

Figure S6. Effect of nucleotides on the formation of higher oligomeric species. Mass photometry profile for WT SpNDK (top and middle panels) and H121A mutant (bottom panels) with gaussian peaks and histograms shown for the different oligomeric states. The percentage of each oligomeric state is also indicated. The proteins were preincubated for 5 min. in the presence of the nucleotide indicated in the figure.

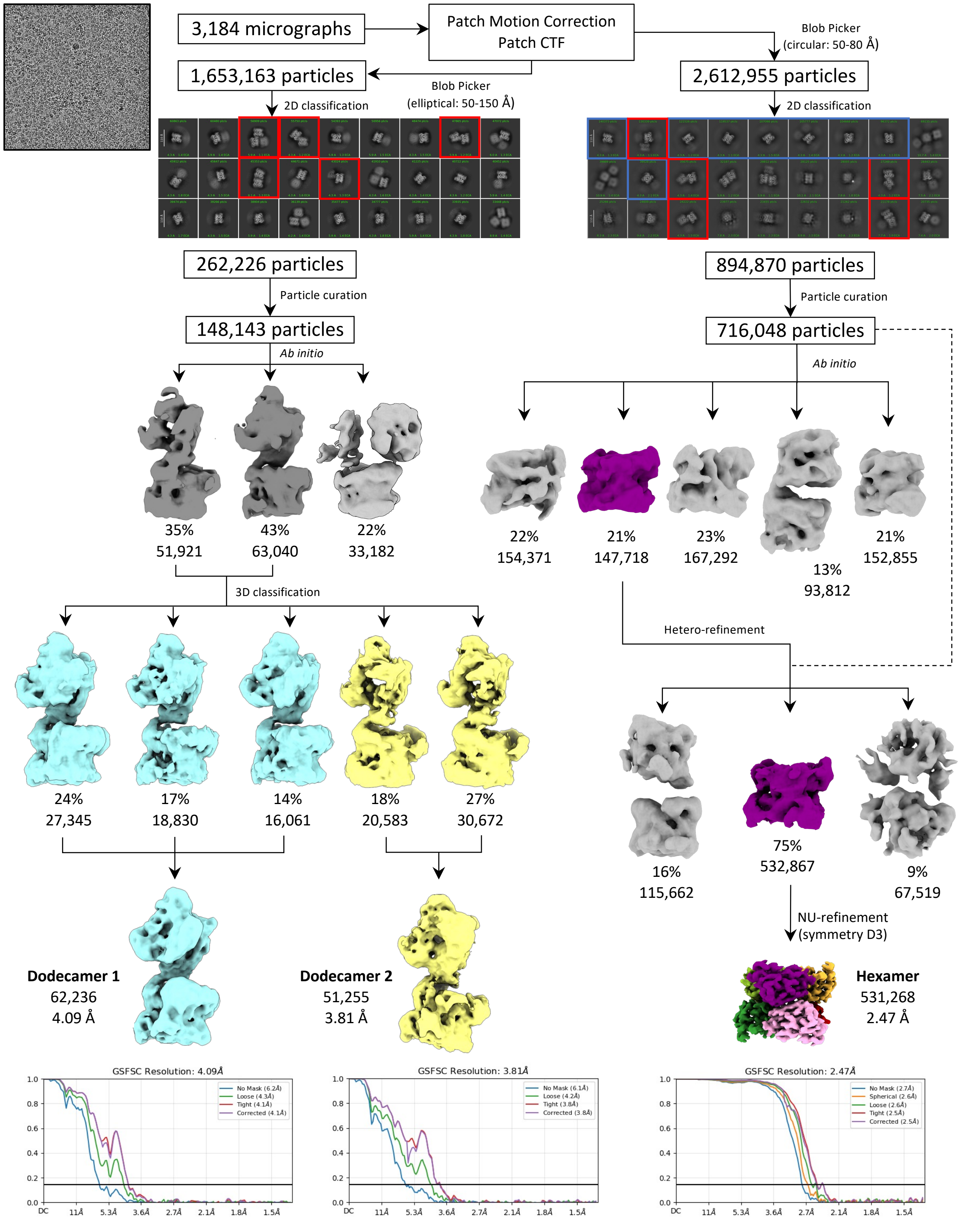

Figure S7. Cryo-EM processing workflow for NDK hexamer and dodecamers. After patch motion correction and CTF estimation, hexameric NDK particles were picked with a circular blob picker. Following rounds of 2D classification, five *ab initio* models were generated, with the best map serving as reference for heterogenous refinement. The resulting hexamer map was further refined with D3 symmetry, yielding a final map at 2.47 Å resolution ($FSC_{0.143}$). For dodecameric NDK, particles were picked with an elliptical blob picker. After two rounds of 2D classification, three *ab initio* maps were generated. A subsequent 3D classification produced two conformations (dodecamer 1 and 2), which were refined separately using non-uniform refinement to 4.09 Å and 3.81 Å resolution ($FSC_{0.143}$).

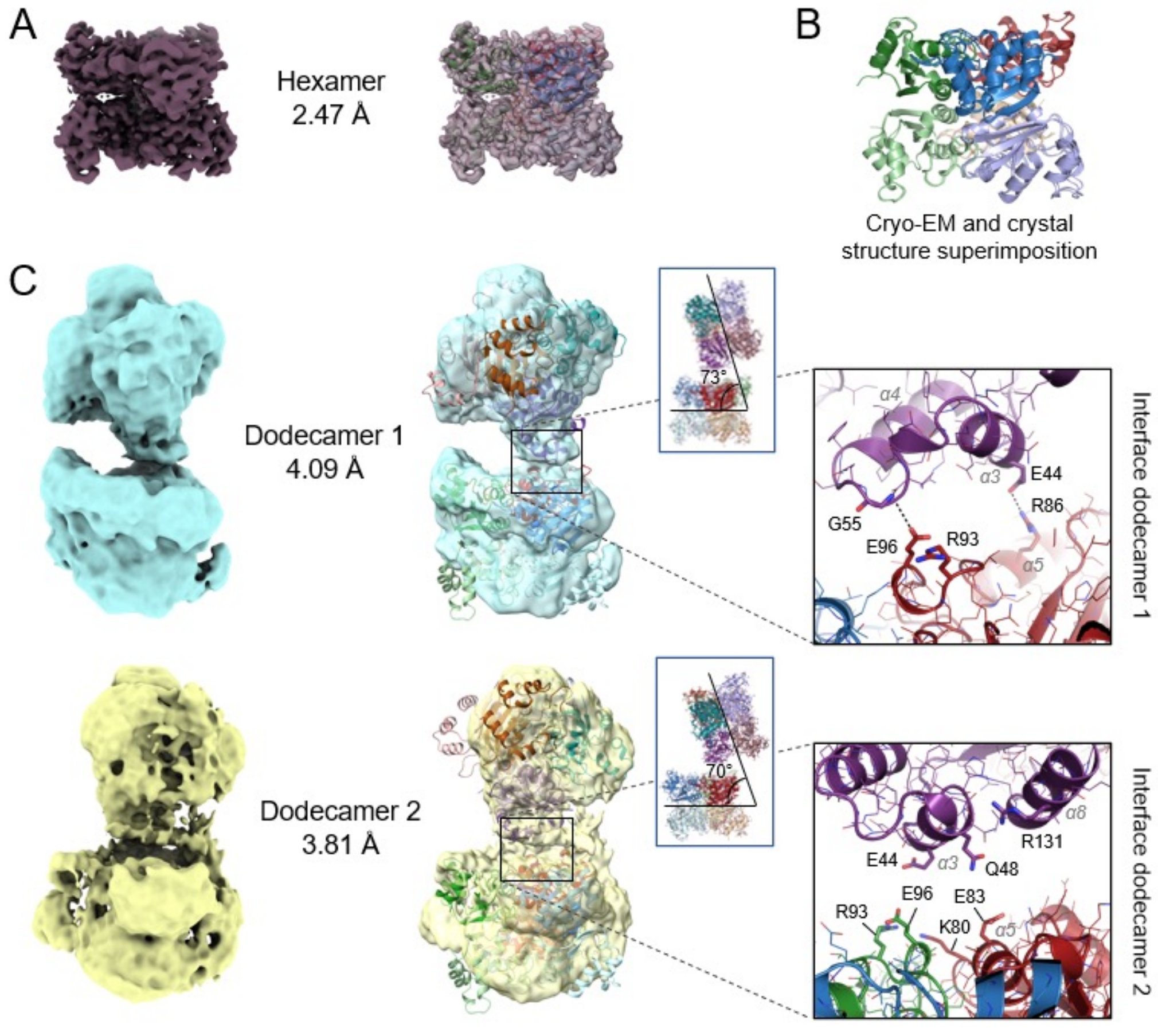

Figure S8. Cryo-EM structures of spNDK. A, Cryo-EM map and model fitting of the hexameric NDK. Each monomer is represented in a different colour. **B**, Superimposition of the cryo-EM model (9SLP) with the crystal structure (9SFO) of hexameric NDK, displaying very high similarity (rmsd of 0.364 over 125 C_{α}). **C**, Cryo-EM maps and model fitting of the hexameric NDK (9SLP) into dodecamer map 1 (top) and 2 (bottom). Relative position of each NDK hexamer within each dodecamer is shown in insert and a close-up view highlighting residues at the interface between two monomers is displayed on the right. Due to the low resolution of the maps, models could not be refined; residue side chains are therefore as observed in the hexameric form.

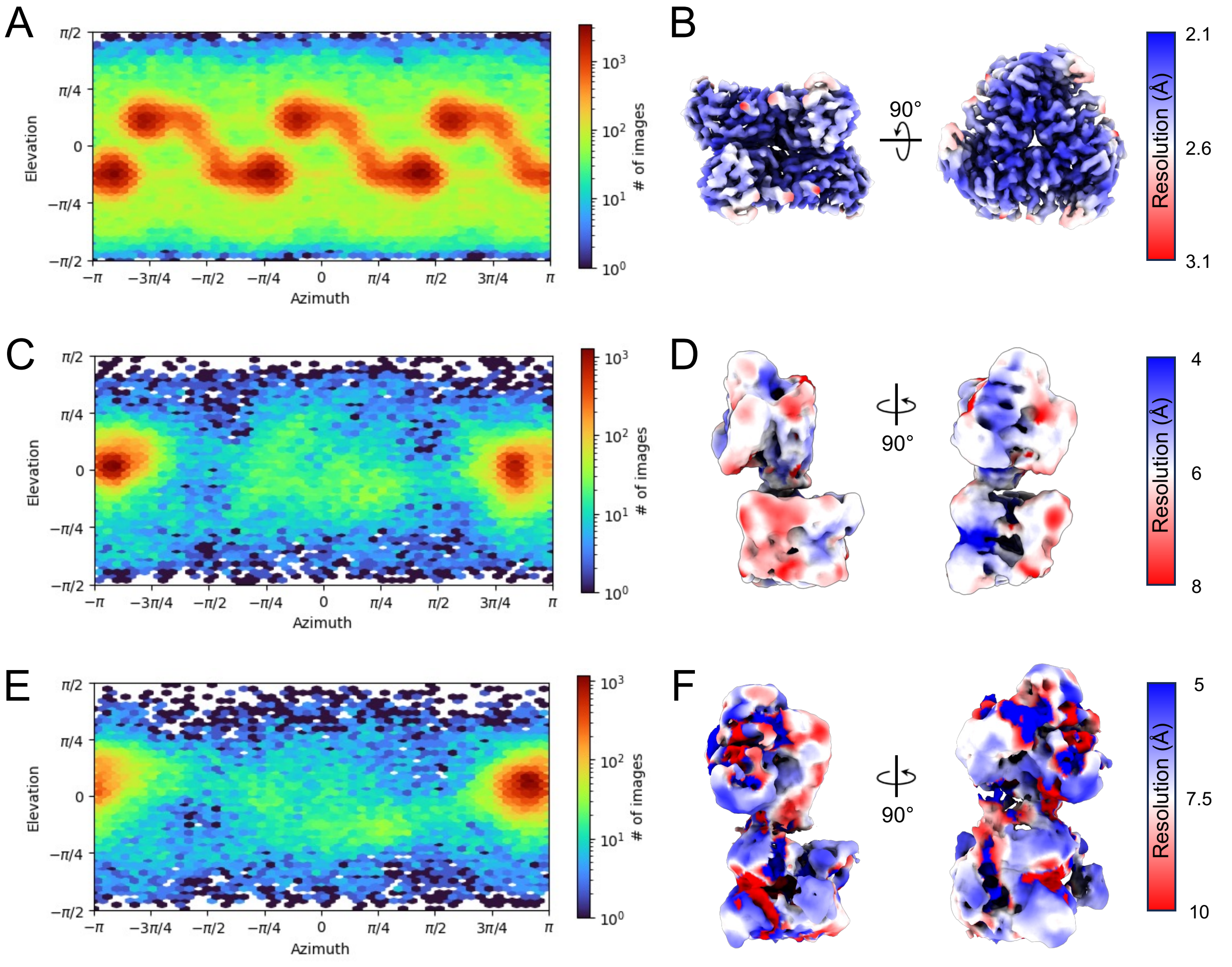

Figure S9. Cryo-EM analysis of NDK. Viewing direction distribution plots of particles for NDK hexamer (**A**), dodecamer 1 (**C**) and dodecamer 2 (**E**). Most frequent views are colored in red. Final cryo-EM map of NDK hexamer (**B**), dodecamer 1 (**D**) and dodecamer 2 (**F**) colored according to the local resolution.
