# Supplementary material for: Quaternary structures of Streptococcus pneumoniae nucleoside diphosphate kinase: From hexamers to supramolecular assemblies
Source: Protein Sci. 2026 Aug 8;35(9):e70735. doi: 10.1002/pro.70735 (PMC13451967; doi:10.1002/pro.70735)
Supplement: Supplementary file 2 [file PRO-35-e70735-s003.docx]

**Table S1. X-ray data and refinement statistics of SpNDK.**

|  | **NDK Apo**  9RVW | **NDK-ADP-vanadate**  9SFO |
| --- | --- | --- |
|  |  |  |
| **Data Collection** |  |  |
| Beamline | ESRF ID30A-3 | ESRF ID23-2 |
| Wavelength (Å) | 0.9677 | 0.8731 |
|  |  |  |
| **Crystal parameters** |  |  |
| Space Group | H 3 2 | P4_1_ 2_1_ 2 |
| Unit Cell Dimensions (Å) | 68.9, 68.9, 156.4 | 199.0, 199.0, 72.6 |
| Unit Cell Angles (°) | 90, 90, 120 | 90, 90, 90 |
| Mosaic Spread (°) | 0.23 | 0.42 |
|  |  |  |
| **Reflection Data** |  |  |
| Resolution Range (Å) | 34.44-1.20 (1.22-1.20) | 72.28-3.42 (3.69-3.42) |
| Unique Reflections | 44110 (1749) | 20087 (4041) |
| *R_sym_* | 0.100 (0.942) | 0.352 (0.929) |
| I/σ(I) | 13.5 (2.0) | 4.5 (2.2) |
| CC½ | 0.994 (0.794) | 0.914 (0.802) |
| Completeness (%) | 98.1 (79.4) | 99.7 (99.8) |
| Multiplicity | 10.4 (7.0) | 5.8 (6.1) |
| Wilson B (Å^2^) | 10.1 | 60.3 |
|  |  |  |
| **Refinement** |  |  |
| Resolution (Å) | 34.46 (1.20) | 70.40 (3.42) |
| Number of Reflections | 41954 | 18978 |
| *R_work_* | 0.1724 | 0.2040 |
| *R_free_* | 0.1853 | 0.2688 |
| Rms (Bond Length) (Å) | 0.014 | 0.004 |
| Rms (Bond Angles) (Å) | 2.023 | 1.449 |
|  |  |  |
| **Model Composition** |  |  |
| Protein atoms | 1088 | 6588 |
| Waters | 174 | / |
| Other | / | 133 |
|  |  |  |
| **Model B-factors** |  |  |
| Protein atoms (Å^2^) | 14.1 | 65.5 |
| Waters (Å^2^) | 26.0 | / |
| Other | / | 83.8 |
|  |  |  |
| **Ramachandran Statistics** |  |  |
| Favoured (%) | 97.0 | 95.0 |
| Allowed (%) | 3.0 | 5.0 |
| Outliers (%) | 1.1 | 0.0 |

Values in parentheses indicate the outer resolution bin. Reflection data and refinement statistics are as reported by Aimless [18] and REFMAC5 [19], respectively. Ramachandran statistics are from Rampage [20].

**Table S2. Cryo-EM data collection and refinement statistics for spNDK.**

|  | | **NDK hexamer**  PDB 9SLP / EMD-55011 | | **NDK dodecamer 1**  EMD-55334 | | **NDK dodecamer 2**  EMD-55333 | |
| --- | --- | --- | --- | --- | --- | --- | --- |
|  | |  | |  | |  | |
| **Data Collection and Processing** |  | |  | |  | |  |
| Microscope | TFS Glacios | | TFS Glacios | | TFS Glacios | |  |
| Camera | TFS Falcon 4i | | TFS Falcon 4i | | TFS Falcon 4i | |  |
| Energy Filter | Selectris X | | Selectris X | | Selectris X | |  |
| Magnification | 165k | | 165k | | 165k | |  |
| Voltage (kV) | 200 | | 200 | | 200 | |  |
| Electron exposure (e^-^/Å^2^) | 59.5 | | 59.5 | | 59.5 | |  |
| Defocus range (µm) | -0.5 to -1.7 | | -0.5 to -1.7 | | -0.5 to -1.7 | |  |
| Pixel size (Å) | 0.694 | | 0.694 | | 0.694 | |  |
| Symmetry imposed | D3 | | C1 | | C1 | |  |
| Initial particle images (no.) | 2,612,955 | | 1,653,163 | | 1,653,163 | |  |
| Final particle images (no.) | 531.268 | | 62.236 | | 51.255 | |  |
| Map resolution (Å) | 2.47 | | 4.09 | | 3.81 | |  |
| FSC threshold | 0.143 | | 0.143 | | 0.143 | |  |
| Map resolution range (Å) | 2.138 - 33.080 | | 1.448 - 64.756 | | 1.448 - 59.343 | |  |
|  |  | |  | |  | |  |
| **Refinement** |  | |  | |  | |  |
| Model resolution (Å) | 2.59 | |  | |  | |  |
| FSC threshold | 0.5 | |  | |  | |  |
| Map sharpening B factor (Å^2^) | 86.97 | |  | |  | |  |
|  |  | |  | |  | |  |
| **Model Composition** |  | |  | |  | |  |
| Non-hydrogen atoms | 6522 | |  | |  | |  |
| Protein residues | 822 | |  | |  | |  |
| Other | 0 | |  | |  | |  |
|  |  | |  | |  | |  |
| **R.m.s. deviations** |  | |  | |  | |  |
| Bond lengths (Å) | 0.002 | |  | |  | |  |
| Bond angles (°) | 0.442 | |  | |  | |  |
|  |  | |  | |  | |  |
| **Validation** |  | |  | |  | |  |
| MolProbity score | 1.24 | |  | |  | |  |
| Clashscore | 4.07 | |  | |  | |  |
| Poor rotamers (%) | 0 | |  | |  | |  |
|  |  | |  | |  | |  |
| **Ramachandran Statistics** |  | |  | |  | |  |
| Favoured (%) | 97.8 | |  | |  | |  |
| Allowed (%) | 2.2 | |  | |  | |  |
| Outliers (%) | 0 | |  | |  | |  |
|  | |  | |  | |  | |
